# ADAM17, induced by Augmenter of Liver Regeneration via G protein-coupled receptor activation, transactivates epidermal growth factor-receptor and reduces classical IL-6 signaling

**DOI:** 10.1186/s12964-026-02782-7

**Published:** 2026-03-07

**Authors:** Christoph Voigt, Sophie Menzel, Rania Dayoub, Marion Kubitza, Florian Schmutzler, Christa Buechler, Elke Eggenhofer, Michael Melter, Thomas S. Weiss

**Affiliations:** 1 Children’s University Hospital (KUNO), University Hospital Regensburg, Regensburg, 93053 Germany; 2 Department of Internal Medicine I, University Hospital Regensburg, Regensburg, 93053 Germany; 3 Department of Surgery, University Hospital Regensburg, Regensburg, 93053 Germany

**Keywords:** ALR, GPCR, ADAM17, EGF-R, IL-6 signaling, Regeneration, Inflammation

## Abstract

**Background:**

Liver regeneration is orchestrated by various cytokines and growth factors, and any imbalance in this process may contribute to liver disease development. Augmenter of Liver Regeneration (ALR), an anti-apoptotic and anti-inflammatory co-mitogen supports regeneration, yet the molecular mechanisms by which ALR regulates proliferation and inflammation remain poorly understood.

**Methods:**

Hepatoma cell lines, primary mouse and human hepatocytes, and mice subjected to ischemia–reperfusion injury, were treated with recombinant ALR. Various specific inhibitors, small interfering RNA, immunoprecipitation, Western blot, qRT-PCR and ELISA techniques were utilized to analyze the underlying signaling pathways.

**Results:**

ALR induces the phosphorylation of the EGF-receptor (EGF-R), which subsequently activates the MAPK and PI3K/Akt pathways. EGF-R phosphorylation is triggered by EGF-R ligands, such as TGFα, amphiregulin and HB-EGF, which are released from plasma membranes by the sheddase a disintegrin and metalloproteinase 17 (ADAM17) upon ALR activation. Furthermore, ALR-activated ADAM17 cleaves the membrane-tethered IL-6-receptor α (gp80), thereby reducing IL-6-induced STAT3 phosphorylation and the expression of its target genes (e.g. ICAM-1) in vitro and in vivo. The induction of ADAM17 involves the phosphorylation of protein kinase C (PKC) and the tyrosine kinase Src, as well as the activation of a G protein-coupled receptor (GPCR) by ALR. ALR transduction across plasma membranes is achieved by activating a Gα_q/11_-coupled GPCR, which is known to induce ADAM17 via cytosolic relay molecules PKC and Src.

**Conclusion:**

Activation of ADAM17 by ALR: i) transactivates EGF-R signaling upon release of membrane-bound EGF-R ligands, and ii) attenuates classical IL-6 signaling upon gp80 shedding. ALR supports liver regeneration by inducing EGF-R-dependent proliferative (MAPK) and anti-apoptotic (PI3K/Akt) pathways, and reduces IL-6-induced inflammatory gene expression.

**Supplementary Information:**

The online version contains supplementary material available at 10.1186/s12964-026-02782-7.

## Introduction

The liver possesses the remarkable capacity to regenerate in response to tissue damage or loss, such as ischemia–reperfusion injury (IRI), toxic injury, infection or partial hepatectomy [1]. Insufficient or dysregulated regeneration following acute injury has been identified as a potential cause of liver fibrosis, cirrhosis and hepatocellular carcinoma [1, 2]. Therefore, it is imperative that this multifactorial process be adequately regulated [1]. A plethora of growth factors and cytokines modulate hepatic proliferation, apoptosis and metabolic processes, including interleukin 6 (IL-6), tumor necrosis factor (TNF), hepatocyte growth factor (HGF) and epidermal growth factor receptor (EGF-R) ligands [3]. A rather novel factor that is known for its hepatoprotective properties and is released upon liver tissue damage is Augmenter of Liver Regeneration (ALR), an anti-apoptotic, anti-oxidative co-mitogen [4, 5].

ALR is constitutively expressed in hepatocytes and cholangiocytes, with localization to both mitochondria and the cytosol [5]. In humans, three distinct isoforms have been identified: two longer forms (21 and 23 kDa) are present in the mitochondria and the cytosol, and a shorter 15 kDa isoform is restricted to the cytosol [5] and is secreted following liver tissue damage [4]. This short form ALR has demonstrated hepatoprotective effects in various liver injury models, including anti-apoptotic, proliferative, and anti-lipotoxic actions (reviewed in [5]). Furthermore, ALR was shown to attenuate IL-6/STAT3-mediated acute phase signaling [6] and to activate downstream EGF-R pathways, including PI3K/Akt and MAPK [7, 8]. The functional relevance of ALR is evidenced by studies showing that ALR knockout is embryonically lethal, and that hepatocyte-specific deletion leads to early-onset of steatosis, mitochondrial dysfunction, and hepatocellular carcinoma [9]. Despite mounting evidence of its hepatotrophic functions, the precise molecular mechanisms by which ALR supports liver regeneration remain to be fully elucidated.

IL-6 is a pleiotropic cytokine, mainly synthesized by monocytes, macrophages, fibroblasts and endothelial cells, which is secreted during inflammatory conditions [10]. It is a pivotal factor in initiating liver regeneration by inducing hepatocyte proliferation [10, 11], but excessive activation of IL-6 signaling can lead to chronic inflammation and tissue damage, such as fibrosis, in conditions such as hepatic IRI [12, 13]. While IL-6 and other cytokines initiate regeneration, growth factors, such as EGF-R ligands, drive proliferation [1, 14]. EGF-R is a transmembrane receptor with intrinsic tyrosine kinase activity that is highly expressed on hepatocytes. Upon ligand binding, EGF-R autophosphorylates carboxyterminal tyrosine residues to initiate signaling by pathways such as MAPK and PI3K/Akt, which in turn regulate proliferation and differentiation [15]. The essential role of EGF-R in liver regeneration is demonstrated in EGF-R-deficient livers, where regeneration is significantly impaired following a two-thirds hepatectomy [16]. Furthermore, EGF-R ligands such as transforming growth factor alpha (TGF-α), amphiregulin (AREG) and heparin-binding EGF-like growth factor (HB-EGF) elicit strong mitogenic responses in cultured hepatocytes.

The protein expression levels of these EGF-R ligands, along with ADAM17 (A Disintegrin And Metalloproteinase 17), undergo a rapid increase following partial hepatectomy and the onset of regeneration [15]. ADAM17, also referred to as TNF-α-converting enzyme (TACE), is a member of a family of endopeptidases that plays a crucial role in the release of membrane-embedded proteins. The ADAM17 protease has been classified as a sheddase due to its function in cleaving the ectodomain of various substrates, thereby regulating membrane fusion (adhesion molecules), growth factors (e.g., TGF-α, HB-EGF, AREG), and cytokine release, as well as shedding of receptor proteins, including IL-6 receptors and tumor necrosis factor receptor α, which are crucial in determining cellular fate and proliferation [17]. Notably, mice lacking ADAM17 exhibit a distinctive developmental phenotype related to EGF-R signaling [18]. In the liver, the most significant substrates of ADAM17 include EGF-R ligands, the IL-6 receptor α, and TNF and its receptors. The proteolytic cleavage of these substrates regulates both proliferative and inflammatory pathways during liver damage and regeneration [19].

The objective of this study is to elucidate the molecular mechanisms by which ALR exerts its proliferative, anti-apoptotic and anti-inflammatory actions, thereby mitigating damage upon IRI. In the following analysis, we examine the mechanisms by which ALR transduces signaling in hepatocytes, whilst concurrently regulating EGF-R- and IL-6-mediated pathways. It is demonstrated that ALR, by activation of a G-protein coupled receptor (GPCR) as well as of the cytosolic relay molecules protein kinase C (PKC) and Src, induces ADAM17. This in turn, transactivates the EGF-R and attenuates classical IL-6 signaling, thereby modulating both proliferative and inflammatory pathways that are important for liver regeneration.

## Material & methods

### Cell culture and treatments

The human hepatoma cell lines HepG2 (HB-8065) and Hep3B (HB-8064) were obtained from the American Type Culture Collection (ATCC, Manassas, VA, USA), and Huh7 cells (ECACC 01042712) from the European Collection of Authenticated Cell Cultures (ECACC, Salisbury, UK). Cells were cultivated at 37 °C, 5% CO_2_ in MEM (HepG2, Hep3B) or RPMI (Huh7), purchased from Thermo Fisher Scientific (Darmstadt, Germany), supplemented with penicillin (100 U/ml), streptomycin (100 µg/ml) and 10% fetal calf serum (Sigma-Aldrich, Taufkirchen, Germany). The cells were seeded at a density of 5 × 10^4^ cells/cm^2^ (HepG2) or 3 × 10^4^ cells/cm^2^ (Hep3B, Huh7), unless otherwise stated, and cultured for 24 h. Thereafter, the cells were starved overnight (18–24 h) in serum-free conditions before being treated as indicated. Recombinant short form ALR was prepared as described elsewhere [20]. The following reagents were used: EGF (10 ng/ml) (Biomol, Hamburg, Germany); IL-6 (25 ng/ml) (PeproTech, Hamburg, Germany); anti-EGF-R antibody clone 225 (10 µg/ml) (Biozol, Eching, Germany); anti-TGFα (1 µg/ml), anti-AREG (1 µg/ml), anti-HB-EGF (1 µg/ml) antibody, (Bio-Techne, Wiesbaden, Germany). Specific inhibitors used in the study: AG1478 (10 µM), Marimastat (20 µM) (both Santa Cruz, Heidelberg, Germany); BIM46187 (5 µM), eCF506 (1 µM), (both Biomol, Hamburg, Germany); GW280264X (20 µM) (Tocris Bioscience, Bristol, UK); Phorbol-12-myristat-13-acetat (PMA; 20 nM), GI254023X (20 µM), NSC-87877 (100 µM), TPI-1 (10 µg/ml) (all Sigma-Aldrich, Taufkirchen, Germany); PD98059 (25 µM) (Bio-Techne, Wiesbaden, Germany); BIMX/Ro-31–8425 (5 µM) (Hycultec, Beutelsbach, Germany). All inhibitors were dissolved in DMSO. The culture medium containing 0.5% DMSO was employed as the solvent control.

### Mouse liver tissue

Mouse liver samples were obtained from a previous study inducing hepatic IRI in an in vivo mouse model without (control PBS) or with application of recombinant ALR [20]. All animal experiments were approved by the institutional committee of Animal Care and Use, University of Regensburg (54 ± 2532.1–2/12; Government of the Upper Palatinate, Germany) and conducted in accordance with the German federal law regarding the protection of animals and 'Guide for the Care and Use of Laboratory Animals' (National Institutes of Health publication 8th Edition, 2011). See the supplementary methods for more details.

### Primary hepatocytes

Primary mouse hepatocytes were isolated from wild-type C57BL/6 (B6) mice, purified, seeded onto collagen-coated culture dishes and were then maintained in culture, as previously described [20]. Primary human hepatocytes (PHH) were isolated from human liver tissue obtained from liver resections of patients undergoing partial hepatectomy for metastatic liver tumors of colorectal cancer and maintained in culture as described elsewhere [21]. See the supplementary methods for more details.

### Cytosol and membrane fraction

The isolation of membrane and cytosolic fractions was conducted using the Mem-PER Plus Membrane Protein Extraction Kit (Thermo Fisher Scientific, Darmstadt, Germany), in accordance with the manufacturer's instructions.

### Western blot analysis

Harvested cells from cell culture experiments were homogenized in 100 µl RIPA (150 mM NaCl, 1.0% Triton-X-100, 0.1% SDS, 50 mM Tris, pH 7.2) buffer containing protease and phosphatase inhibitors (Roche, Penzberg, Germany) and 5 mM EDTA for a period of 15 min prior to centrifugation (16,000 g for 20 min). Western blot analysis was performed according to standard protocols. Briefly, total protein lysates (20 µg per lane) were separated by 12% SDS-PAGE (Bio-Rad, Hercules, CA, USA) under reducing conditions, transferred onto 0.2 µm polyvinylidene difluoride membranes (Fisher Scientific, Schwerte, Germany), incubated overnight with specific antibodies (for details see supplementary Table S1) and developed with ECL reactions (Bio-Rad, Hercules, CA, USA). Densitometric data were generated for Western blots using Image Lab (Bio-Rad, Hercules, CA, USA), with the results given in the respective figures. All samples utilized for western blot analysis were obtained from independent biological replicates. For a comprehensive overview of the densitometric data, summary statistics, and statistical testing, please refer to the supplementary Table S3.

### Immunoprecipitation

The cells were lysed in RIPA buffer (Carl Roth, Karlsruhe, Germany) containing protease and phosphatase inhibitors (Roche, Penzberg, Germany) and 5 mM EDTA for a period of 15 min prior to centrifugation (16,000 g for 20 min). Thereafter, 700 µg of protein lysate was incubated with 3.5 µg of an anti-EGF-R antibody (clone 528; Biozol, Eching, Germany) at 4 °C for 18 h. 50 µl of protein A agarose beads were washed 4 times with PBS before incubation with immune complexes for 3 h at 4 °C. Subsequently, the beads were subjected to three washes in PBS, two washes in PBS containing 0.5% Triton X-100, and were then analyzed by SDS-PAGE and Western Blot.

### Enzyme-linked immunosorbent assay (ELISA)

HepG2 cells were seeded at a density of 2.5 × 10^4^ cells/cm^2^, cultured for 24 h and starved overnight in serum free conditions. For sgp80 and sgp130 analysis, cells were pretreated with Marimastat (60 min) before application of ALR or PMA for 24 h. For sICAM-1 analysis, cells were pretreated with ALR (100 nM, 1 h) before application of IL-6 for 12 h or 24 h. Commercially available ELISA kits were used to determine the release of sgp80, sgp130 (Invitrogen, ThermoFisher Scientific, Darmstadt, Germany) and sICAM-1 (Abcam, Cambridge, UK) in cell culture supernatants according to manufacturers’ instructions.

### Transfection of cells with siRNA

For the siRNA transfection, HepG2 cells were grown in 6-well plates (0.2 × 10^6^ cells per well). The cells were then transfected with 25 pmol of GNAQ siRNA (s5887; Thermo Fisher Scientific, Darmstadt, Germany), ADAM17 siRNA (sc-36604), SHP1 siRNA (sc-29478) or a scrambled control siRNA (sc-37007; all from Santa Cruz Biotechnology, Heidelberg, Germany) for sequence-independent effects. The transfection was performed in the presence of Lipofectamine RNAiMAX reagent (Thermo Fisher Scientific, Darmstadt, Germany) according to the manufacturer's instructions. The transfected cells were cultured for 48 h and then treated with ALR and IL-6, as indicated. The cells were then harvested and prepared for further analysis.

### RNA isolation, reverse transcription and qRT-PCR

Total RNA was isolated from cells using the RNeasy Mini Kit (Qiagen, Hilden, Germany). First strand cDNA was synthesized from 1 µg of total RNA using the Quantitect Reverse Transcription Kit (Qiagen, Hilden, Germany). The mRNA levels of ADAM10, ADAM17, FGB, HAMP, HP, ICAM-1, SAA2, TIMP3, mgp80, mgp130, sgp130, total gp130 and HPRT1 (primer sequences are listed in supplementary Table S2) were quantified by real-time PCR technology using the LightCycler 480 SYBR Green I Master mix (Roche, Penzberg, Germany) according to the manufacturer’s instructions. PCR reaction products were verified by sequence analysis and each PCR analysis was performed in triplicate.

### Statistical analysis

All data are presented as mean ± standard deviation as indicated. Data were compared between groups using Student’s t-test or ANOVA with post-hoc Bonferroni correction where appropriate. Values of *p* < 0.05 were considered significant (SPSS Statistics 25.0 program, IBM, Leibniz Rechenzentrum, München, Germany).

## Results

### ALR activates MAPK- and PI3K/Akt-pathways via EGF-R signaling

**Fig. 1:**
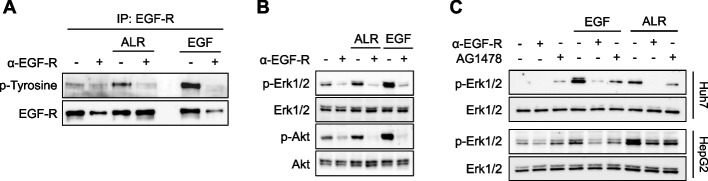
ALR induces EGF-R phosphorylation, thereby activating MAPK- and PI3K/Akt-pathways dependent on EGF-R-ligand binding and EGF-R-tyrosine kinase activity. Hep3B cells were pre-incubated for 60 min in the absence or presence of an EGF receptor antibody (α-EGF-R clone 225, 10 µg/ml). Following this, the cells were treated with EGF (10 ng/ml) or ALR (100 ng/ml) for a further 20 min. **A** Cell homogenates were immuno-precipitated with an anti-EGF-R antibody (α-EGF-R clone 528), separated by SDS-PAGE, and immunoblotted with a phospho-tyrosine antibody. **B** The aforementioned Hep3B cells were subjected to immunoblotting for Erk1/2 and Akt phosphorylation (*n* = 3: Hep3B, *n* = 1, Huh7, *n* = 2). **C** Huh7 and HepG2 cells were pre-incubated without or with EGF receptor antibody (α-EGF-R clone 225, 10 µg/ml, 60 min) or the EGF-R-tyrosine kinase inhibitor AG1478 (10 µM, 30 min). The cells were then treated with EGF (10 ng/ml) or ALR (100 ng/ml) for 20 min and immunoblotted for Erk1/2 phosphorylation (*n* = 4–6: HepG2, *n* = 1, Hep3B, *n* = 2, Huh7, *n* = 3). Further Information regarding replicates, densitometric and statistical analysis can be found in supplementary Table S3

It has been demonstrated that ALR activates MAPK and PI3K/Akt pathways, which are known to promote regeneration and cell growth [8]. The present study therefore sought to investigate the impact of ALR on the activation of the EGF-R, and its subsequent effect on the MAPK and PI3K/Akt pathways. Hep3B cells treated with ALR (recombinant short form ALR) demonstrated increased phosphorylation of EGF-R tyrosine residues (Fig. 1A), Erk1/2 and Akt (Fig. 1B) in comparison to untreated cells. Inhibition of EGF- (positive control) and ALR-induced phosphorylation of EGF-R and its downstream effectors Erk1/2 and Akt was observed upon pre-incubation with a blocking anti-EGF-R antibody (α-EGF-R) (Figs. 1A-C). Furthermore, the inhibition of EGF-R tyrosine kinase activity by treatment with AG1478 diminished EGF- and ALR-induced phosphorylation in Huh7 and HepG2 cells (Fig. 1C). It can be concluded that both the binding of EGF-R-ligands to its receptor and the activity of EGF-R tyrosine kinase are essential for the induction of the MAPK and PI3K/Akt pathways by ALR.

### ALR induces MAPK- and PI3K/Akt-pathways in response to activation of Gα_q/11_ coupled GPCR and Src

**Fig. 2:**
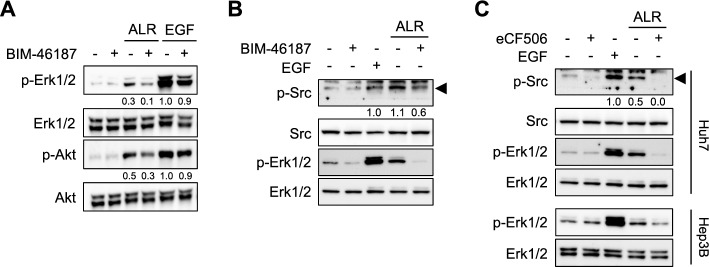
ALR induces MAPK- and PI3K/Akt-pathways in response to GPCR activation and Src phosphorylation. **A** Hep3B cells, pre-incubated without or with GPCR inhibitor BIM46187 (5 µM, 30 min), were treated with EGF (10 ng/ml) or ALR (100 ng/ml) for 20 min and immunoblotted for Erk1/2 (*n* = 3: HepG2, *n* = 1, Hep3B, *n* = 1, Huh7, *n* = 1) and Akt phosphorylation. **B** Huh7 cells, pre-incubated without or with GPCR inhibitor BIM46187 (5 µM, 30 min), were treated with EGF (10 ng/ml) or ALR (100 ng/ml) for 20 min and immunoblotted for Erk1/2 and Src phosphorylation. (p-Erk1/2 in A and B, *n* = 3: HepG2, *n* = 1, Hep3B, *n* = 1, Huh7, *n* = 1) **C** Huh7 and Hep3B cells, pre-incubated without or with Src inhibitor eCF506 (1 µM, 30 min), were treated with EGF (10 ng/ml) or ALR (100 ng/ml) for 20 min and immunoblotted for Src and Erk1/2 phosphorylation (p-Erk1/2, *n* = 4: Hep3B, *n* = 2, Huh7, *n* = 2; p-Src, *n* = 2). Densitometric analysis was performed in **A**) on Erk1/2 and Akt, in **B**) and **C**) on Src phosphorylation (ratio phospho/non-phospho) and normalized to EGF-treated cells. Further information regarding replicates, densitometric and statistical analysis can be found in supplementary Table S3

**Fig. 3:**
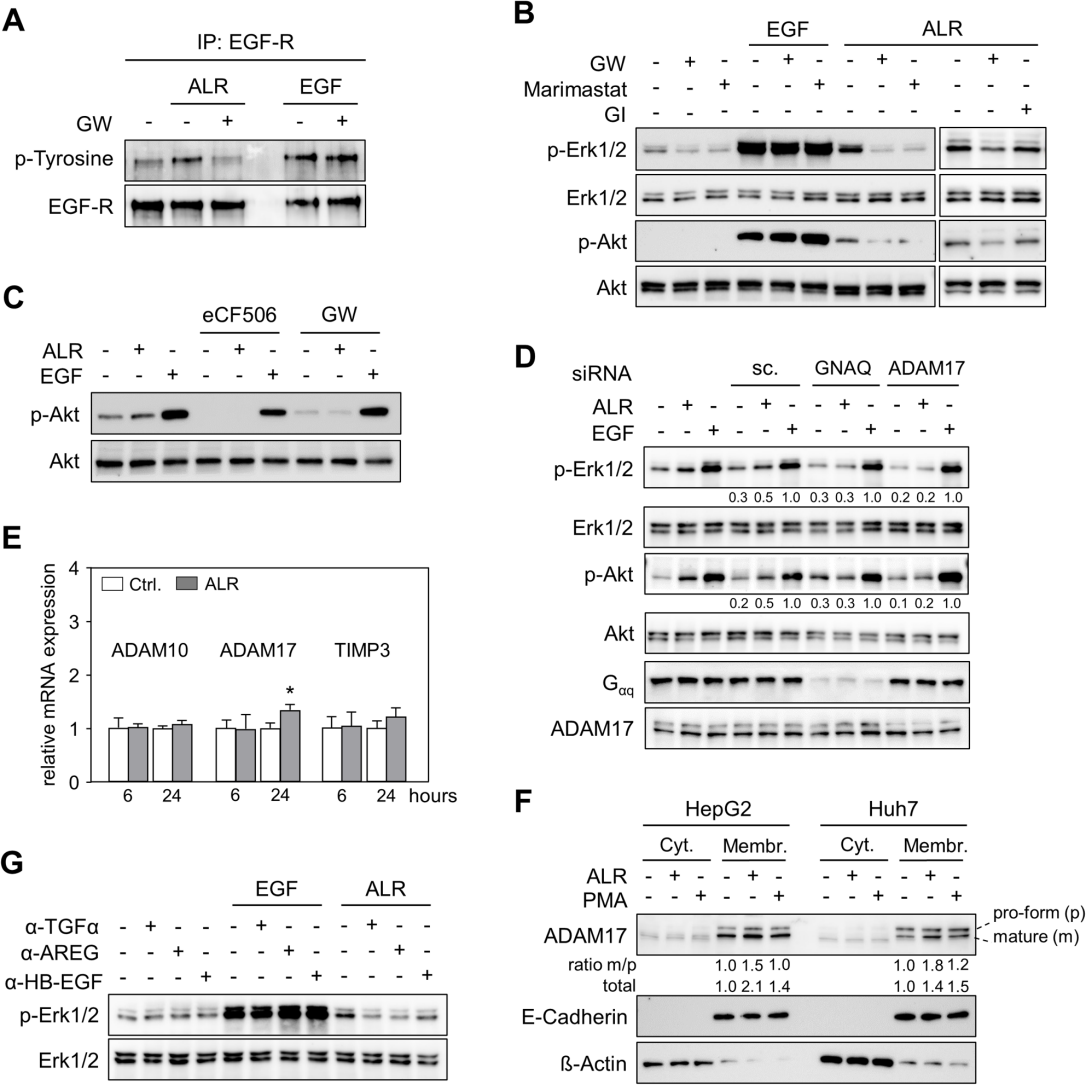
ALR triggers sheddase ADAM17, followed by release of membrane-bound EGF-R-ligands and activation of EGF-R downstream signaling. **A** Hep3B cells, pre-incubated without or with sheddase ADAM10/ADAM17 inhibitor GW (GW280264X, 20 μM,30 min), were treated with EGF (10 ng/ml) or ALR (100 ng/ml) for 20 min. Subsequent to this, cell homogenates were immuno-precipitated with an anti-EGF-R antibody (α-EGF-R clone 528),separated by SDS-PAGE and immunoblotted with a phospho-tyrosine antibody. **B** Hep3B cells were pre-incubated without or with ADAM10/ADAM17 inhibitor GW (20 μM, 30 min),ADAM/MMP inhibitor Marimastat (20 μM, 30 min) or ADAM10 inhibitor GI (GI254023X, 20 μM,30 min), and were treated with EGF (10 ng/ml) or ALR (100 ng/ml) for 20 min and immuno blotted for Erk1/2 and Akt phosphorylation (p-Erk1/2, n=3: Hep3B, n=2, HepG2, n=1; p-Akt, n=2: Hep3B, n=1, HepG2, n=1). **C** Primary mouse hepatocytes isolated from three distinct mouse livers (M1 – M3) were pre-incubated without or with Src inhibitor eCF506 (1 μM, 30 min) or ADAM10/ADAM17 inhibitor GW (20 μM, 30 min), and then treated with EGF (10 ng/ml) orALR (100 ng/ml) for 20 min followed by immunoblotting for Akt phosphorylation. **D** Hep3B cells were pre-incubated without or with GNAQ (Gα), ADAM17 or scrambled control (sc.) siRNA for48 hours, and were treated with EGF (10 ng/ml) or ALR (100 ng/ml) for 20 min. Thereafter, immuno blotting was performed to analyze the phosphorylation of Erk1/2, and Akt. Densitometric analysis was performed on Erk1/2 and Akt and Src phosphorylation (ratiophospho/non-phospho) and normalized to the corresponding EGF-treated cells. **E** HepG2 cells were incubated in the absence or presence of ALR (100 ng/ml) for 6 or 24 h. The mRNA expression levels of sheddases ADAM10 and ADAM17, in addition to their intrinsic inhibitorTIMP3, were analyzed through the utilization of qRT-PCR and subsequently normalized to the house keeping gene HPRT1 (three independent experiments, mean ± SD). * *p*  <  0.05 differs from corresponding untreated control (Ctrl). **F** Huh7 and HepG2 cells were treated either with PMA(20 nM) or ALR (100 ng/ml) for 20 min. Separated cytosolic (Cyt.) and membrane (Membr.) cell fractions were immunoblotted for ADAM17 expression, using E-cadherin and β-actin as controls (n=4: HepG2, n=2, Huh7, n=2). Densitometric analysis was performed to determine the ratio mature to pro-form (m/p) membrane-bound ADAM17 expression, with the total amount ofADAM17 (mature and pro-form) normalized to untreated cells. **G** HepG2 cells were pre-incubated without or with neutralizing antibodies against TGFα (α-TGFα, 1 μg/ml, 30 min), HB-EGF (α-HB-EGF, 1 μg/ml, 30 min) or AREG (α-AREG, 1 μg/ml, 30 min), and were treated with EGF (10 ng/ml) or ALR (100 ng/ml) for 20 min and immunoblotted for Erk1/2phosphorylation. Further information regarding replicates, densitometric and statistical analys is can be found in supplementary Table S3

Preliminary binding analyses and affinity cross-linking experiments indicated a specific, high-affinity receptor for ALR on rat hepatocytes and HepG2 cells, with a putative receptor size of approximately 75 kDa [22]. The receptor binding activity and specificity of ALR were analyzed by displacement tests, which suggested that there is no direct physical interaction between ALR and the receptors for EGF and TGFα [22]. In addition, it was demonstrated that ALR does not phosphorylate the HGF (c-met) or the IGF-1 receptor. It is known that these two receptors potentially activate the EGF-R and MAPK pathway [7]. Moreover, it has been reported that membrane receptors, including GPCRs, are capable of regulating cell growth via the EGF-R and its associated signaling network [23]. In light of this, we analyzed the transactivation of EGF-R by GPCR upon ALR stimulation. Treatment of hepatoma cells with ALR resulted in increased levels of p-Erk1/2 and p-Akt, which were strongly reduced in the presence of BIM-46187 (Fig. 2A and B), a G protein inhibitor with high selectivity to block Gα_q/11_ activation [24]. The involvement of Gα_q/11_ activation was further substantiated by silencing Gα_q/11_ expression (supplementary Fig. S2A), which blunted ALR-induced Erk1/2 and Akt phosphorylation (Fig. 3D). Although treatment with BIM-46187 appears to result in slightly lower p-Erk1/2 levels in EGF (Fig. 2A) or control treated cells (Fig. 2B), the reason for this remains unclear. Furthermore, this effect was not observed in the other cell lines used (Fig. 2A, B, supplementary Fig. S1A). Overall, ALR treatment led to an increase in both p-Erk1/2 and p-Akt, which was reduced upon the addition of BIM-46187. Moreover, GPCR blockade by BIM-46187 reduced phosphorylation of the cytosolic non-receptor tyrosine kinase Src (Fig. 2B), which is a hallmark of EGF-R transactivation by GPCR [23]. The involvement of Src activation in ALR-induced EGF-R transactivation is demonstrated by treating various hepatoma cells with a Src-specific inhibitor, eCF506, leading to non-phosphorylated Src and consequently to blunted p-Erk1/2 signals (Fig. 2C, supplementary Fig. S1B). It is notable that EGF-induced Erk1/2 phosphorylation was found to be dependent of EGF-R tyrosine kinase activity (Fig. 1C), but independent of Src inhibition (supplementary Fig. S1B), which points to an involvement of Src activation by ALR upstream of EGF-R. The transactivation of MAPK and PI3/Akt pathways by ALR requires the involvement of GPCR, most likely coupled to Gα_q/11_ and the activation of non-receptor tyrosine kinase Src.

### ALR activates sheddase ADAM17 and consequently release of EGF-R-ligands inducing EGF-R and its downstream signaling

Ligand-dependent EGF-R transactivation implies the activation of Src targets with proteolytic activity, such as ADAMs, as well as the liberation of their membrane-bound substrates, e.g. EGF-R ligands. In order to investigate this further, Hep3B cells were treated with ALR or EGF in the absence or presence of inhibitors for ectodomain shedding proteins ADAM10/17 with GW280264X (GW), ADAM10 with GI254023X (GI), or metalloproteinases with Marimastat. The results demonstrated that the phosphorylation of Erk1/2 and Akt by ALR was significantly reduced by Marimastat and GW, but not by GI (Fig. 3B). In line with this, GW blocked ALR-stimulated EGF-R activation, as evidenced by reduced phosphorylation of EGF-R tyrosine residues (Fig. 3A). The activation of EGF-induced EGF-R and the subsequent Erk1/2 and Akt phosphorylation were found to be independent of any applied inhibitor (Fig. 3A-B). The ability of ALR to activate EGF-R and subsequently MAPK and PI3K/Akt pathways is not only dependent on GPCR and Src, but also on sheddase ADAM17 activity. This finding is further substantiated by studies conducted on primary mouse hepatocytes, which demonstrate that the inhibition of Src or ADAM17 led to a diminution in ALR-induced Akt phosphorylation, while EGF signaling remained unaltered (Fig. 3C). Furthermore, experiments reducing ADAM17 expression by utilizing the RNA interference technique (supplementary Fig. S2B) confirmed the important role of ADAM17 in ALR signal-transduction, since ALR failed to phosphorylate Erk1/2 and Akt in Hep3B cells with low ADAM17 expression (Fig. 3D). This finding indicates an induction of both Src and ADAM17 by ALR upstream of EGF-R activation (Fig. 3C, D). Furthermore, treatment of hepatoma cells with ALR resulted in a modest enhancement of ADAM17 mRNA expression, while constitutively expressed ADAM10 remained unaltered (Fig. 3E) and tissue inhibitor of metalloproteinases 3 (TIMP3), a negative regulator of ADAM17 activity [25], was not changed at the mRNA (Fig. 3E) or protein level (supplementary Fig. S3). Of significance, the induction of ADAM17 activity by ALR was substantiated by the augmented levels of ratios of mature to pro-form ADAM17 in membrane fractions of cells treated with ALR (Fig. 3F). Concurrently, there was a moderate increase in the expression of total ADAM17 in membrane fractions (Fig. 3F). PMA, a known ADAM17 sheddase activator, was utilized as a control and enhanced ADAM17 activity, exhibiting only marginal alteration in its protein abundance on plasma membranes [26]. In addition, the study examined whether the shedding of EGF-R ligands, subsequent to enhanced ADAM17 activity, is relevant to EGF-R activation. This was achieved through antibody-mediated depletion of the EGF-R ligands TGFα, AREG or HB-EGF in culture media (Fig. 3G). Treatment of cells with ALR revealed no induction of the MAPK pathway (Erk1/2 phosphorylation) in the presence of antibodies against TGFα, AREG and, to a lesser extent, HB-EGF. In contrast, the MAPK pathway induced by EGF remained unaffected (Fig. 3G). In summary, ALR activates a GPCR, which in turn leads to the phosphorylation of cytosolic Src, induction and activation of the sheddase ADAM17, followed by the release of membrane-bound EGF-R ligands and their subsequent intracellular signaling. This sequence is described as a triple-membrane-passing mechanism responsible for ligand-dependent EGF-R transactivation.

### ALR decreases IL-6-induced STAT3 phosphorylation independent of EGF-R, SHP1 or SHP2 activity

**Fig. 4:**
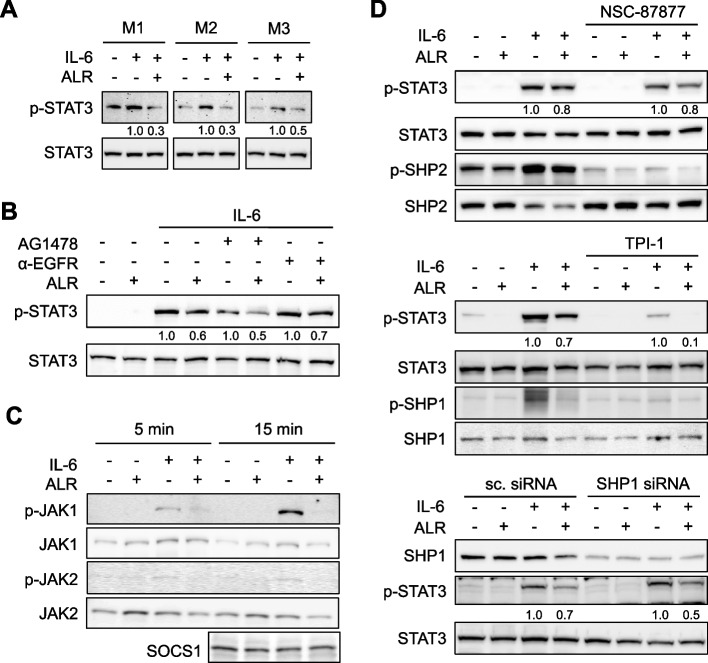
ALR attenuates IL-6-induced STAT3 phosphorylation independent of EGF-R activity and JAK regulating phosphatases SHP1 or SHP2. **A** Primary mouse hepatocytes (obtained from three mouse livers, *n* = 3) were pre-incubated without or with ALR (100 ng/ml, 60 min), then treated with IL-6 (25 ng/ml) for 15 min and immunoblotted for STAT3 phosphorylation. **B** Hep3B cells, pre-incubated without or with an anti-EGF-R antibody (α-EGF-R clone 225, 10 µg/ml, 90 min) or an EGF-R-tyrosine kinase inhibitor AG1478 (10 µM, 90 min) and ALR (100 ng/ml, 60 min), were treated with IL-6 (25 ng/ml) for 15 min and immunoblotted for STAT3 phosphorylation (*n* = 2). **C** HepG2 cells, pre-incubated without or with ALR (100 ng/ml, 60 min), were treated with IL-6 (25 ng/ml) for 5 and 15 min and immunoblotted for p-JAK1, JAK1, pJAK2, JAK2 and SOCS1 expression. **D** Cells, pre-incubated without or with ALR (100 ng/ml, 60 min) and SHP1/2 inhibitor NSC-87877 (100 µM, 90 min; Huh7, *n* = 2) or SHP1 inhibitor TPI-1 (HepG2, 10 µg/ml, 90 min; *n* = 3; HepG2, *n* = 2, Huh7, *n* = 1) or SHP1 siRNA (48 h; HepG2, *n* = 2), were treated with IL-6 (25 ng/ml) for 15 min and immunoblotted for SHP2 or SHP1 and STAT3 phosphorylation. Densitometric analysis was performed for STAT3 phosphorylation (ratio p-STAT3/STAT3) and normalized to corresponding IL-6-treated cells without ALR treatment. Further information regarding replicates, densitometric and statistical analysis can be found in supplementary Table S3

As indicated by previous research, treatment with ALR has been shown to reduce IL-6-induced signal transduction [6]. Therefore, we investigated whether ALR may affect IL-6 signaling by activating EGF-R or regulating factors of IL-6 signaling. The present study has confirmed and expanded upon previous findings, demonstrating a decrease in p-STAT3, the major downstream signal of IL-6 receptor (IL-6R) activation, in hepatoma cells (supplementary Fig. S4) and primary mouse hepatocytes following ALR treatment and IL-6 induction (Fig. 4A). In order to exclude a cross-reactivity with activated EGF-R signaling, cells were treated with either a blocking anti-EGF-R antibody or an EGF-R inhibitor, AG1478. Reduced STAT3 phosphorylation was still observed upon ALR treatment and IL-6 induction (Fig. 4B). Furthermore, ALR treatment did not alter the expression of the feedback inhibitor suppressor of cytokine signaling (SOCS) 3 and the negative feedback loop protein inhibitor of activated STAT 3 (PIAS3) (data not shown [6]), nor negative regulator SOCS1 (Fig. 4C). Phosphorylation of Janus kinases JAK1 and JAK2, which testify the activation of IL-6R (dimerization of IL-6R subunits gp80 and gp130), was blunted in ALR treated cells upon IL-6 induction (Fig. 4C). IL-6R activation at this stage can be negatively regulated by de-phosphorylation of JAK1/2 through protein tyrosine phosphatases SHP1 or SHP2 [27]. Therefore, we examined their participation in ALR reduced IL-6 signaling (Fig. 4D). The inhibition of SHP1 and SHP2 by specific inhibitors (TPI, NSC-87877), as indicated by the absence of their phosphorylation, did not prevent ALR from reducing p-STAT3 levels. This was further confirmed by silencing SHP1 expression, demonstrating the irrelevance of SHP1 on the reduction of STAT3 activity by ALR upon IL-6 stimulation (Fig. 4D).

We next asked whether decreased phosphorylation of JAK1/2, and therefore less activated IL-6R complex, was related to IL-6R subunit expression levels. Treatment of cells with ALR did not alter the mRNA expression of membrane-bound IL-6Rα (mgp80) or IL-6Rβ (mgp130), nor soluble sgp130 or total gp130 (supplementary Fig. S5A). ALR treatment also did not alter the phosphorylation of gp130 at Ser782 (supplementary Fig. S5B), indicating internalization and degradation of gp130. In addition, we performed FACS analysis, but found no difference in cell surface expression of gp130 and gp80 upon ALR treatment (data not shown), with the latter being poorly expressed on hepatoma cells. In conclusion, while ALR reduces IL-6-induced p-STAT3, independent of EGF-R, PIAS, SOCS1/3 and SHP1/2, ALR does not alter the regulation of IL-6R expression.

### ALR attenuates IL-6-induced STAT3 phosphorylation by activation of sheddase ADAM17 dependent on Gα_q/11_ coupled GPCR, Src and PKC

**Fig. 5:**
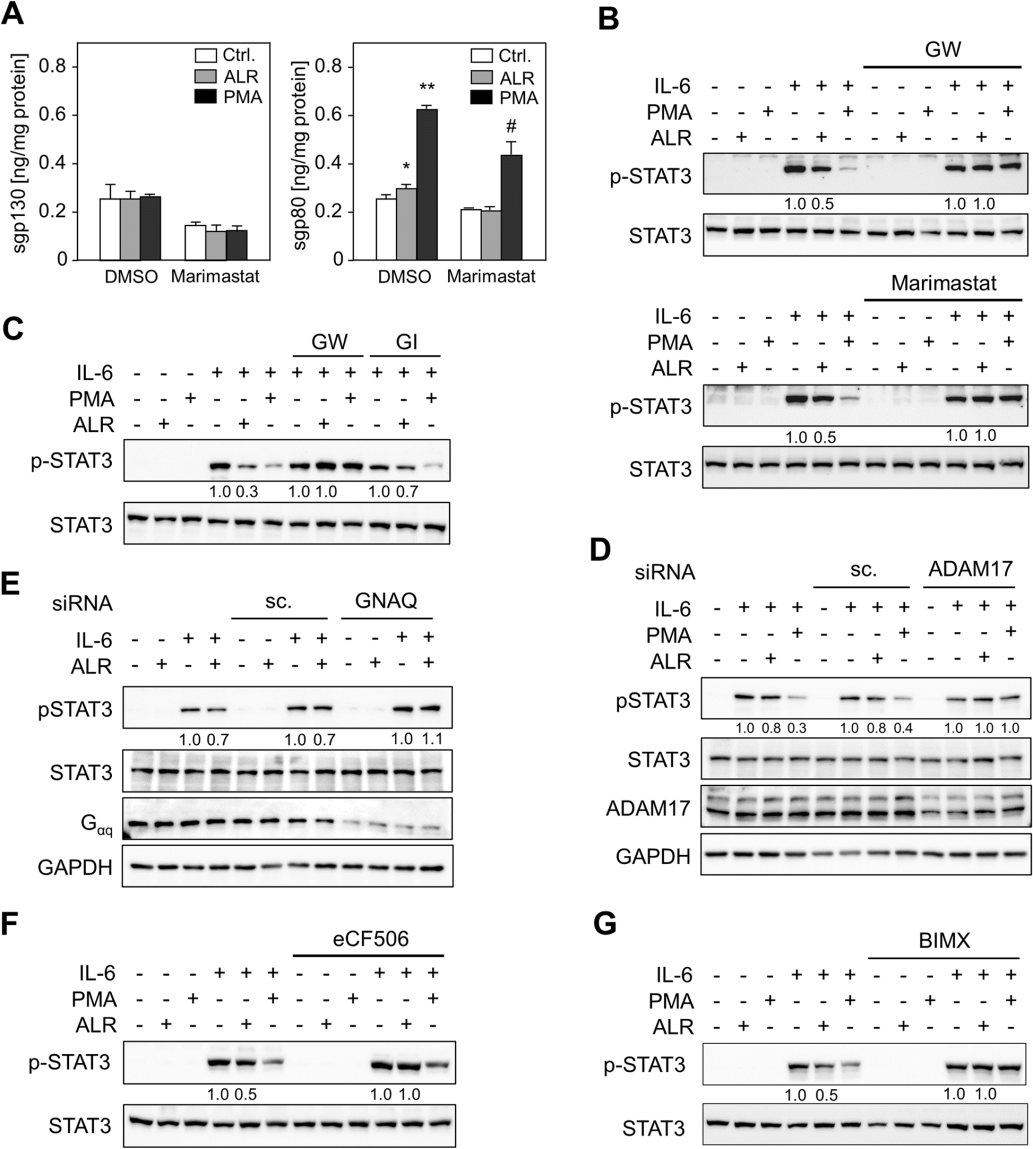
ALR attenuates IL-6-induced STAT3 phosphorylation by activation of sheddase ADAM17 dependent on Src and PKC. **A** HepG2 cells, pre-incubated without or with ADAM/MMP inhibitor Marimastat (20 µM, 60 min), were treated with PMA (20 nM) or ALR (100 ng/ml) for 24 h and levels of soluble gp130 (sgp130) and gp80 (sgp80) released into the culture medium were determined by ELISA (three independent experiments, mean ± SD). * *p* < 0.05 and ** *p* < 0.01 differs from untreated control (Ctrl), # *p* < 0.05 differs from corresponding PMA treated cells. **B** Hep3B cells were pre-incubated without or with ADAM10/ADAM17 inhibitor GW (GW280264X, 20 µM, 90 min) or ADAM/MMP inhibitor Marimastat (20 µM, 90 min), PMA (20 nM, 10 min) or ALR (100 ng/ml, 60 min). The cells were then treated with IL-6 (25 ng/ml) for 15 min and immunoblotted for STAT3 phosphorylation (*n* = 3: Hep3B, *n* = 1, HepG2, *n* = 1, Huh7, *n* = 1). **C** HepG2 cells were pre-incubated without or with GW (20 µM, 90 min) or ADAM10 inhibitor GI (GI254023X, 20 µM, 90 min), and PMA (20 nM, 10 min) or ALR (100 ng/ml, 60 min). The cells were then treated with IL-6 (25 ng/ml) for 15 min and immunoblotted for STAT3 phosphorylation (*n* = 2). **D** HepG2 cells were subjected to 48 h of pre-incubation in the absence or presence of ADAM17 or a scrambled control (sc.) siRNA. The cells were then exposed to PMA (20 nM, 10 min) or ALR (100 ng/ml, 60 min) and subsequently were treated with IL-6 (25 ng/ml) for 15 min and immunoblotted for STAT3 phosphorylation (*n* = 2). Immunoblotting for GAPDH functions as a loading control. **E** HepG2 cells were subjected to 48 h of pre-incubation in the absence or presence of GNAQ (Gα_q_) or scrambled control (sc.) siRNA. The cells were then exposed to PMA (20 nM, 10 min) or ALR (100 ng/ml, 60 min) and subsequently were treated with IL-6 (25 ng/ml) for 15 min and immunoblotted for STAT3 phosphorylation (*n* = 2). Immunoblotting for GAPDH functions as a loading control. HepG2 cells were pre-incubated without or with (**F**) Src inhibitor eCF506 (1 µM, 90 min; *n* = 3: HepG2, *n* = 1, Hep3B, *n* = 1, Huh7, *n* = 1) or (**G**) pan-PKC inhibitor BIMX (Ro31-8425, 5 µM, 90 min; *n* = 2: HepG2, *n* = 1, Hep3B, *n* = 1), and PMA (20 nM, 10 min) or ALR (100 ng/ml, 60 min). Following this, the cells were treated with IL-6 (25 ng/ml) for 15 min and immunoblotted for STAT3 phosphorylation. Densitometric analysis was performed for STAT3 phosphorylation (ratio of p-STAT3/STAT3) and normalized to corresponding IL-6-treated cells without ALR or PMA incubation. Further information regarding replicates, densitometric and statistical analysis can be found in supplementary Table S3

The shedding protease ADAM17 has been demonstrated to have various substrates, including growth factors, cytokines, adhesion molecules and receptors [17, 19]. It is therefore reasonable to hypothesize that the release of IL-6Rα by shedding is implicated in the reduced IL-6 signaling observed in response to ALR. To test this hypothesis, the culture media of cells treated with ALR or PMA, a protein sheddase activator, were analyzed. This analysis revealed an increased release of soluble gp80 (sgp80), which was blocked by the metalloproteinase inhibitor marimastat (Fig. 5A). The levels of sgp130 remained unaltered by both treatments, which is attributed to the all-transcriptional regulation of sgp130 [28]. Furthermore, we treated various hepatoma cell lines in the absence or presence of inhibitors for sheddases ADAM10/17 (GW) or metalloproteinases (Marimastat) with ALR and IL-6 (Fig. 5B, supplementary Fig. S6). Our results showed that the application of ALR led to a reduction in IL-6-induced STAT3 phosphorylation, while PMA completely blocked this process. In addition, GW or Marimastat treatment prevented both of these effects. To further refine these results, the ADAM10/17 inhibitor GW was utilized in comparison to the ADAM10 inhibitor GI within the same experimental framework (Fig. 5C). Once more, the treatment of cells with ALR or PMA led to a reduction in IL-6-induced p-STAT3 levels, which were blocked by GW, but not by GI (Fig. 5C). This finding was further corroborated by the demonstration that there was no reduction of IL-6-induced STAT3 phosphorylation by ALR or PMA, while there was reduced ADAM17 expression upon addition of ADAM17 siRNA (Fig. 5D, supplementary Fig. S2B). This finding underscores the pivotal role of ADAM17 in this process.

Furthermore, evidence is presented demonstrating the dependence of the alleviation of IL-6 induced p-STAT3 by ALR on Gα_q/11_ coupled GPCR and Src activity. The reduction of Gα_q/11_ expression by siRNA technique (supplementary Fig. S2A) abrogated the diminishing effect of ALR on IL-6-induced STAT3 phosphorylation (Fig. 4E) and the enhancement of p-STAT3 was retained upon the inhibition of Src by eCF506 (Fig. 4F). Furthermore, the study revealed that the reducing effect of ALR and PMA, the latter of which is known to induce PKC, on IL-6-induced p-STAT3 levels was blocked by BIMX (Ro31-8425), thus indicating that PKC is also part of ALR-mediated signaling (Fig. 4G). Conclusively, the findings indicate that ALR treatment results in a reduction in IL-6 signaling, a process which is contingent on the activation of sheddase ADAM17, as well as Src and PKC. This is a characteristic feature of Gα_q/11_ coupled GPCR-induced ADAM17 activity and, consequently, is responsible for receptor shedding, as demonstrated for release of sgp80 and reduced IL-6 signaling.

### ALR reduces p-STAT3 regulated target genes, attenuates members of the inflammatory response and thereby mitigates ischemia reperfusion injury

**Fig. 6:**
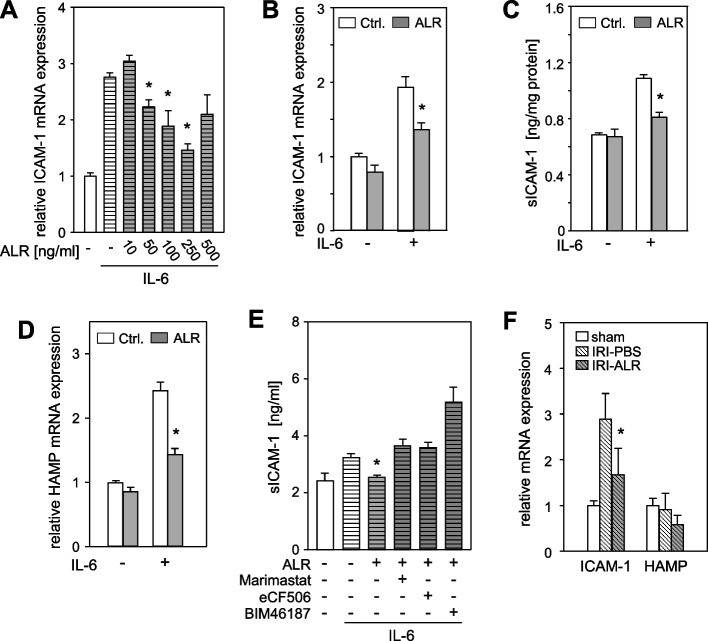
ALR reduces IL-6-induced ICAM-1 and hepcidin expression and thereby attenuates tissue damage in a mouse model of hepatic ischemia reperfusion injury. **A** HepG2 cells and **B** Primary human hepatocytes were incubated with IL-6 (25 ng/ml) for 24 h and pre-treated without or with A) increasing concentrations of ALR and B) 100 ng/ml ALR, for 60 min followed by qRT-PCR for ICAM-1 mRNA expression analysis. **C** HepG2 cells, treated as in B), were analyzed for soluble ICAM-1 (sICAM-1) released into the culture medium by ELISA. **D** HepG2 cells, treated as in B), were analyzed for iron regulating hepcidin (HAMP) mRNA expression by qRT-PCR. Data in A), B), and D) have been normalized to HPRT1. **E** HepG2 cells, pre-incubated without or with ALR (100 ng/ml, 60 min) and Src inhibitor eCF506 (1 µM, 90 min), ADAM/MMP inhibitor Marimastat (10 µM, 90 min) or GPCR inhibitor BIM46187 (5 µM, 90 min), were treated with IL-6 (25 ng/ml) for 12 h and levels of soluble ICAM-1 (sICAM-1) released into the culture medium were determined by ELISA. A through E: three independent experiments, mean ± SD, * *p* < 0.05 different from corresponding IL-6 treated cells). **F** In a model of hepatic IRI, mice were treated with ALR (100 µg/kg b.w.) at 1 h prior to the induction of ischemia (1.5 h) and at the onset of reperfusion (3 h) (*n* = 5). Liver tissues obtained from IRI mice after reperfusion were analyzed for ICAM-1 and hepcidin mRNA expression and normalized to HPRT1 (mean ± SD). * *p* < 0.05 different from IRI-PBS-treated mice

Activation of STAT3 upon IL-6 induction has been identified as a regulator of a variety of target genes, including those that are critical to the inflammatory response, such as the acute phase proteins and the intercellular adhesion molecule 1 (ICAM-1) [29]. In the present study, the impact of ALR treatment on IL-6/STAT3 target gene expression was investigated. The analysis revealed a reduction in fibrinogen β (FGB), haptoglobin (HP), serum amyloid A2 (SAA2) (supplementary Fig. S7A) and ICAM-1 mRNA expression (Fig. 6A) in response to different concentrations of ALR prior to IL-6 induction in hepatoma cells. This ALR-mediated reduction of IL-6 target gene expression was further substantiated in primary human hepatocytes (Fig. 6B, supplementary Fig. S8). In addition, a reduction in soluble ICAM-1 (sICAM-1) levels was observed in the culture media of IL-6- and ALR-treated cells in comparison to IL-6 alone (Fig. 6C). The absence of an ALR-mediated reduction of IL-6-induced sICAM-1, in conjunction with the inhibition of ADAM17, Src, or GPCR, serves to reinforce the proposed signal transduction of ALR (Fig. 6E). It is noteworthy that the diminution of IL-6/STAT3 target gene expression by ALR, as demonstrated by acute phase proteins, is not dependent on EGF-R or Erk1/2 (MAPK) pathway activation (supplementary Fig. S7B). Furthermore, an additional STAT3 target gene, hepcidin (*HAMP*), was analyzed. The hepcidin protein is responsible for iron homeostasis and is involved in ferroptosis and inflammation. This analysis revealed that IL-6-triggered *HAMP* mRNA expression was lower upon ALR treatment in vitro (Fig. 6D).

ALR has been shown to attenuate tissue damage in a hepatic IRI mouse model, reducing neutrophil infiltration and oxidative stress [20]. Furthermore, ALR-treated animals exhibited reduced expression of chemo-attractants, such as CXCL2 and CCL2 [20], which have been identified as IL-6/STAT3 target genes [30]. In addition to these observations, we report here enhanced mRNA expression of ICAM-1, a pivotal adhesion molecule during leukocyte recruitment, in IRI livers, which is reduced by ALR treatment (Fig. 5F). This may provide a further explanation for the reduced levels of neutrophils observed in IRI livers treated with ALR [20].

## Discussion

Liver injury, such as that caused by ischemia–reperfusion, can elicit tissue regeneration, driven by the availability of cytokines and growth factors that contribute to inflammation and the promotion or prevention of cell death. The process is intricate and comprises multiple pathways. Membrane-tethered proteases, including ADAM17, have been shown to integrate inflammatory and growth-related pathways [31]. ALR, known to be released during liver injury, has previously been demonstrated to have anti-apoptotic, anti-oxidative and anti-inflammatory properties [5]. Although its relevance in liver physiology and liver regeneration is undisputed, the mechanism behind its action remained obscure. The present study demonstrates that ALR activates the sheddase ADAM17, which in turn releases membrane-bound EGF-R ligands, thereby transactivating EGF-R known to mediate survival and anti-apoptotic signaling. Secondly, ADAM17 sheds IL-6Rα, thereby attenuating IL-6-induced STAT3 target gene expression, leading to reduced ICAM-1 expression in vitro and in vivo. The induction of ADAM17 is dependent on PKC and Src, which are phosphorylated upon activation of a Gα_q/11_ coupled GPCR by ALR (supplementary Fig. S9).

The existing knowledge on the signal transduction-induced anti-apoptotic and proliferative mechanisms of ALR responsible for promoting liver regeneration is incomplete [5]. A previous report demonstrated the activation of EGF-R and MAPK by ALR in HepG2 cells, excluding the binding of EGF to the EGF-R. This finding suggested the existence of a ligand-independent EGF-R pathway [7]. Notably, EGF-R can be transactivated by both ligand-dependent and -independent mechanisms, resulting in enhanced cell proliferation, a process that is facilitated by GPCRs [32]. We present several lines of evidence that ALR activates ligand-dependent EGF-R signaling by inducing the release of the EGF-R ligands AREG, HB-EGF and TGFα. Ligand-independent EGF-R activation is thought to result in incomplete downstream signaling, with e.g. only MAPK induction, whereas EGF-R ligand-dependent signaling leads to the simultaneous activation of MAPK and Akt pathways [32, 33], as has been seen in our study. Furthermore, ligand-dependent EGF-R signaling has been specifically attributed to Gα_q/11_ of the heterotrimeric G-protein family [33, 34] and the involvement of Gα_q/11_ in GPCR-mediated signaling of ALR is further substantiated in this study through the utilization of the GPCR inhibitor BIM-46187 [24] and silencing of Gα_q/11_ expression.

Activation of GPCRs is known to induce mitogenic pathways [35] leading to cell growth, for instance of hepatocytes by TGFα [3]. The activation of mitogenic pathways by GPCRs is diverse and attributed to indirect or co-mitogen growth-related factors [3], the characteristics of which are exhibited by ALR [5, 36]. Although ALR induces the proliferation of hepatoma cells and hepatocytes [5], it has been reported not to stimulate DNA synthesis in isolated rat hepatocytes [37] and some non-hepatic cells [8]. Otherwise, induction of cell proliferation by ALR was observed in parallel with EGF-R or Erk1/2 blockage [8]. Furthermore, Kupffer cells that had been treated with ALR demonstrated enhanced expression of IL-6 and TNF, which are well-known for their cell cycle priming activities, and the activation of a high-affinity cholera-toxin sensitive (Gα_s_-coupled) receptor was initiated by ALR [37]. These findings, which are diverse in nature, along with the ability of GPCRs to interact with several different G-proteins, serve to emphasize the high degree of complexity that is associated with GPCR signaling [33, 35]. It becomes evident that GPCR signaling is cell-specific, receptor-specific and G-protein-type specific, as well as dependent on the cellular network [33]. Consequently, the identification of the type of GPCR that ALR engages and that is coupled to Gα_q/11_ transactivating EGF-R on hepatocytes or coupled to Gα_s_ activating Kupffer cells is of importance and requires further examination.

GPCR transactivation is characterized by a molecular diversity in the mediation of downstream signals (transactivation pattern). A notable example of this is the classical ligand-dependent transactivation of the EGF-R by GPCRs, which occurs via a triple-membrane-passing signaling (TMPS) pathway [33, 38], where the cellular signal has to cross the membrane three times. Thereby, GPCR activation induces various cytosolic relay molecules, which stimulate MMPs or ADAMs to cleave membrane-bound EGF-R ligands and subsequently activates EGF-R signaling pathways. As evidenced in this study for ALR, a classical TMPS pathway is suggested, involving the following: i) a GPCR, ii) the cytosolic tyrosine kinase Src and PKC, iii) the sheddase ADAM17 and iv) EGF-R signaling. The membrane-bound protease, ADAM17, has been shown to cleave not only ligands for EGF-R but also a variety of other membrane proteins, including TNF, TNF-receptor (TNFR1 and TNFR2) and IL-6R. The shedding of these proteins has been identified as being critical in the processes of inflammation and liver injury [19, 39]. As demonstrated by Murthy et al. [31], the hepatocyte-specific ablation of ADAM17 results in increased cell death upon induction of apoptosis, thus highlighting the pivotal role of ADAM17 in liver damage. Furthermore, inactivation of TIMP3, a negative regulator of ADAM17 activity [40], reduced Fas-triggered intrinsic apoptosis, since TIMP3 reduces TNFR shedding and inhibits metalloproteinase-dependent EGF-R signaling [31]. As was demonstrated previously, ALR has the capacity to attenuate apoptosis [21, 41] and to activate Erk1/2 and Akt pathways [8, 41]. This is most likely mediated by ALR-induced activation of ADAM17 and the subsequent shedding of EGF-R ligands, while TIMP3 may not have a role herein (Fig. 3E). Notably, ALR has been observed to reduce the pro-inflammatory cytokines IL-1β and TNF [42, 43], both of which are targets of TNFR signaling. Furthermore, we postulate that the stimulation of ADAM17 by ALR may result in TNFR shedding from hepatocytes and, consequently, a diminution in the activation of NF-κB, a pivotal transcription factor that regulates inflammation and regeneration [44]. In addition, a previous study has demonstrated that ALR has the capability to release TNF from Kupffer cells [37]. Therefore, it is of interest to analyze the potential impact of ALR on TNF signaling in liver cells in future studies.

As previously reported, ALR treatment reduced IL-6-induced phosphorylation of STAT3, which subsequently leads to lower release of fibrinogen β and haptoglobin [6]. The JAK/STAT3 pathway is subject to regulation at multiple levels. This is achieved through the action of physiological antagonists, including PIAS, SOCS and cytoplasmic protein tyrosine phosphatases (PTPs, e.g. SHP1 and SHP2) (reviewed in [27]). ALR, while diminishing IL-6-induced phosphorylation of STAT3, does not affect PIAS3 or SOCS3, as demonstrated previously [6], and did not change SOCS1 levels (Fig. 3). Furthermore, the inhibition of SHP1 and SHP2 activity did not modify the impact of ALR on IL-6-induced p-STAT3 levels, suggesting that these proteins are not responsible for the blunted JAK1/2 phosphorylation observed upon ALR treatment (Fig. 3). In addition, the involvement of SHP2 as a signal mediator of IL-6 induced Erk1/2 activation, which may also regulate the acute phase protein haptoglobin [45], was excluded as a target of ALR by this study. As demonstrated in supplementary Fig. S7B, the inhibition of Erk1/2 with PD98059 does not modify the reduction of IL-6-induced acute phase proteins, namely fibrinogen, haptoglobin and SAA2, by ALR. Moreover, cross-reactivity between IL-6 receptor and EGF-R signaling has been described as a contributing factor to the regenerative process after liver injury, which includes activation of Erk1/2 and STAT3. As demonstrated by Wang [46], the activation of EGF-R was found to attenuate the expression of IL-6-induced acute phase protein (APP) genes haptoglobin and SAA2. In the present study, it was demonstrated that ALR's effect on IL-6-stimulated STAT3 phosphorylation and induction of APP genes is independent of EGF-R signaling. This is evidenced by the inhibition of EGF-R and Erk1/2 (Fig. 3B, Suppl. Fig. S7B).

The induction of IL-6 signaling is initiated by the binding of IL-6 to a membrane-bound non-signal transducing IL-6 receptor α (IL-6Rα, gp80) and the signaling receptor gp130. It has been established that only the complex of IL-6 and IL-6Rα can bind to gp130. In addition, while gp130 is expressed ubiquitously on cells, IL-6Rα is predominantly present on hepatocytes and leukocytes [10, 39]. Upon the formation of the IL-6 signaling complex and its subsequent assembly with gp130, JAKs, associated with the intracellular portion of gp130, undergo auto-phosphorylation [47]. The process of activation is only initiated upon the binding of a ligand. Therefore, the reduction of p-JAK1/2 levels in the presence of ALR upon IL-6 induction is primarily attributable to a decrease in IL-6 receptor availability. The expression of IL-6Rα and gp130 has been observed to be subject to regulation by differential splicing or proteolytic processing of membrane-bound receptors [48]. The present study has demonstrated that the latter is the mediating mechanism through which ALR regulates IL-6 signaling, namely by activating metalloprotease ADAM17. In the process of reducing membrane-bound IL-6Rα and lowering the level of classical IL-6 signaling, a further consequence is the generation of soluble IL-6Rα (sIL-6Rα). This sIL-6Rα, upon formation of complexes with IL-6, is able to bind to gp130 on cells even in the absence of IL-6Rα expression, thereby inducing IL-6 trans-signaling [17, 48]. The expression of IL-6 has been linked not only to the onset of inflammation, but also to protective and regenerative mechanisms. The specific role of classical or IL-6 trans-signaling varies according to the specific cell type and the prevailing cellular environment [17, 19, 39, 48]. Within the liver, classical IL-6 signaling is predominantly an inflammatory response to injury, leading to the induction of APPs. Conversely, IL-6 trans-signaling has been proposed to play a pivotal role in the process of liver regeneration [19, 39]. Therefore, it is of interest to investigate whether the liver regeneration-supporting capabilities of ALR are not only mediated by enhanced EGF-R signaling, but also are connected to enhanced sIL-6R levels, as observed in this study.

In hepatocytes, classical IL-6 signaling comprises STAT3 activation and, consequently, the induction of APPs and chemokines in instances of acute liver injury. Disruption of STAT3 in hepatocytes results in lower hepatic inflammation subsequent to the acute administration of CCl_4_, thereby suggesting an anti-inflammatory role [30, 49]. As demonstrated earlier, serum levels of ALR exhibit a prompt increase in response to liver injury [4] and higher ALR expression was observed in an hepatic IRI model [20]. Furthermore, treatment of IRI mice with ALR reduced hepatic apoptosis and neutrophil infiltration; this phenomenon might be explained by a reduction in the expression of certain hepatocytic chemokines, such as CXCL2 and CCL2 [20]. Both are targets of STAT3 signaling [30], and consequently, we hypothesize that their diminished expression parallels reduced APPs (e.g. fibrinogen) and adhesion molecule ICAM-1 expression upon IL-6 and ALR treatment. It has been demonstrated that a pharmacological reduction of fibrinogen expression and disposition can result in a reduction of platelet aggregation and clot disposition [50]. This, in turn, can lead to a decrease in microvascular injury and hepatocellular apoptosis after hepatic ischemia through early reperfusion. In addition, increased ICAM-1 expression on hepatocytes has been observed in hepatic IRI models. A reduction in ICAM-1 expression is associated with reduced IRI and, moreover, may also attenuate neutrophil migration during parenchymal infiltration [51, 52]. In conclusion, in cases of acute liver injury and high levels of IL-6, ALR has been shown to mitigate IL-6 signaling, thereby reducing STAT3 target genes, including chemokines, APPs and ICAM-1, which consequently might reduce the influx of neutrophils.

Despite presenting convincing evidence of ALR's capacity to activate EGF-R-mediated pathways and modulate IL-6-induced signal transduction, the study is not without limitations. It has been demonstrated that the activation of a Gα_q/11_ coupled GPCR by ALR results in the subsequent activation of ADAM17. However, the specific GPCR to which ALR binds remains to be identified. A significant number of Gα_q/11_-coupled GPCRs are expressed in hepatic cells, and further studies are required to identify which one is activated by ALR. Furthermore, ADAM17 is recognized for its variety of different substrates, of which the consequences of IL-6 trans-signaling on liver regeneration has not yet been investigated. Despite the unfeasibility of statistical testing for all particular Western blot experiments due to the limited number of replications, the results of various experiments that employ Western blot analysis consistently indicate a uniform conclusion. While the focus has been on elucidating the role of ALR in diminishing inflammation, as evidenced by the reduction of neutrophil infiltration, in a IRI mouse model as previously reported [20], further investigations are required in vivo to substantiate the molecular mechanisms of ALR in IRI and other liver regeneration in vivo models, in which ALR has been shown to exert beneficial effects.

## Conclusions

In summary, ALR has been shown to support liver regeneration and diminish cell damage upon liver injury [4, 5, 20, 36, 41]. In this instance, ALR instigates the activation of a Gα_q/11_ coupled G-protein-coupled receptor on hepatocytes, which has the capacity to induce ADAM17. Upon activation, the cell surface protein ADAM17 can liberate membrane-bound EGF-R ligands through shedding. These, in turn, induce EGF-R signaling, leading to the activation of pro-survival pathways, namely Akt and MAPK. Moreover, the activation of ADAM17 by ALR, mediated by Gα_q/11_ coupled GPCR, Src and PKC activation, has been demonstrated to cleave the IL-6Rα, thereby attenuating the expression of IL-6-triggered STAT3 target genes and IL-6-induced inflammation (supplementary Fig. S9). The function of ALR in supporting liver regeneration can be described as follows: ALR enhances the activation of pro-proliferative and anti-apoptotic pathways, thereby attenuating inflammation.

## Supplementary Information

Supplementary Material 1. Supplementary methods.

Supplementary Material 2. Table S1: Primary and secondary antibodies used for Western blotting. Table S2: Primers used in qRT-PCR experiments. Table S3: Densitometric analysis of western blots.

Supplementary Material 3. Figures S1 to S9.

Supplementary Material 4. Legends of Figure S1 to S9. Figure S1: ALR induces Erk1/2 phosphorylation dependent on GPCR and Src activation upstream of EGF-R. Figure S2: Silencing of GNAQ and ADAM17 mRNA expression. Figure S3: Expression of the ADAM17 inhibitor TIMP3 is not induced upon ALR treatment in vitro. Figure S4: ALR attenuates IL-6-induced STAT3 phosphorylation. Figure S5: Administration of ALR does not change mRNA expression of IL-6-receptor subunits αand βnor degradation of gp130 protein. Figure S6: ALR attenuates IL-6-induced STAT3 phosphorylation by activation of sheddase ADAM17 in hepatoma cell lines. Figure S7: ALR reduces IL-6 induced acute phase proteins in hepatoma cells. Figure S8: ALR reduces IL-6 induced acute phase proteins in primary human hepatocytes. Figure S9: Graphical summary.

Supplementary Material 5.

## Data Availability

The datasets used and/or analyzed during the current study are available from the corresponding author on reasonable request.

## Abbreviations

ADAM: A disintegrin and metalloproteinase
ALR: Augmenter of liver regeneration
APP: Acute phase protein
AREG: Amphiregulin
EGF: Epidermal growth factor
EGF-R: EGF-receptor
FGB: Fibrinogen β
Gα: G protein subunit α
GI: GI254023X
gp: Glycoprotein
GPCR: G protein-coupled receptor
GW: GW280264X
HB-EGF: Heparin-binding EGF-like growth factor
HGF: Hepatocyte growth factor
HP: Haptoglobin
ICAM-1: Intercellular adhesion molecule 1
IL-6R: IL-6 receptor
IRI: Ischemia–reperfusion injury
JAK: Janus Kinase
MAPK: Mitogen-activated protein kinases
MMP: Matrix metalloproteinase
PHH: Primary human hepatocytes
PI(3)K: Phosphatidylinositol-4,5-bisphosphate 3 kinase
PIAS: Protein inhibitor of activated STAT
PKC: Protein kinase C
PMA: Phorbol-12-myristat-13-acetat
PTP: Protein tyrosine phosphatase
SAA2: Serum amyloid A2
SH2: Src homology 2
SHP1: Src homology region 2 domain-containing phosphatase 1
SHP2: Src homology region 2 domain-containing phosphatase 2
SOCS: Suppressor of cytokine signaling
STAT: Signal transducer and activator of transcription
TGFα: Transforming growth factor α
TIMP3: Tissue inhibitor of metalloproteinases 3
TNF: Tumor necrosis factor
TNFR: TNF receptor

## References

[1] Michalopoulos GK, Bhushan B. Liver regeneration: biological and pathological mechanisms and implications. Nat Rev Gastroenterol Hepatol. 2021;18(1):40–55. 32764740 10.1038/s41575-020-0342-4

[2] Rodimova S, Mozherov A, Elagin V, Karabut M, Shchechkin I, Kozlov D, et al. Effect of hepatic pathology on liver regeneration: the main metabolic mechanisms causing impaired hepatic regeneration. Int J Mol Sci 2023;24(11):9112. 10.3390/ijms24119112 PMC10252688 37298064

[3] Kimura M, Moteki H, Ogihara M. Role of hepatocyte growth regulators in liver regeneration. Cells. 2023;12(2):208. 10.3390/cells12020208 PMC9856461 36672143

[4] Gandhi CR, Kuddus R, Subbotin VM, Prelich J, Murase N, Rao AS, et al. A fresh look at augmenter of liver regeneration in rats. Hepatology. 1999;29(5):1435–45. 10216127 10.1002/hep.510290522 PMC2978975

[5] Ibrahim S, Weiss TS. Augmenter of liver regeneration: essential for growth and beyond. Cytokine Growth Factor Rev. 2019;45:65–80. 30579845 10.1016/j.cytogfr.2018.12.003

[6] Dayoub R, Buerger L, Ibrahim S, Melter M, Weiss TS. Augmenter of liver regeneration (ALR) exhibits a dual signaling impact on hepatic acute-phase response. Exp Mol Pathol. 2017;102(3):428–33. 28506765 10.1016/j.yexmp.2017.05.011

[7] Li Y, Li M, Xing G, Hu Z, Wang Q, Dong C, et al. Stimulation of the mitogen-activated protein kinase cascade and tyrosine phosphorylation of the epidermal growth factor receptor by hepatopoietin. J Biol Chem. 2000;275(48):37443–7. 10982794 10.1074/jbc.M004373200

[8] Ilowski M, Putz C, Weiss TS, Brand S, Jauch KW, Hengstler JG, et al. Augmenter of liver regeneration causes different kinetics of ERK1/2 and Akt/PKB phosphorylation than EGF and induces hepatocyte proliferation in an EGF receptor independent and liver specific manner. Biochem Biophys Res Commun. 2010;394(4):915–20. 20230786 10.1016/j.bbrc.2010.03.074

[9] Gandhi CR, Chaillet JR, Nalesnik MA, Kumar S, Dangi A, Demetris AJ, et al. Liver-specific deletion of augmenter of liver regeneration accelerates development of steatohepatitis and hepatocellular carcinoma in mice. Gastroenterology. 2015;148(2):379–91. 25448926 10.1053/j.gastro.2014.10.008 PMC4802363

[10] Schmidt-Arras D, Rose-John S. IL-6 pathway in the liver: from physiopathology to therapy. J Hepatol. 2016;64(6):1403–15. 26867490 10.1016/j.jhep.2016.02.004

[11] Taub R. Liver regeneration 4: transcriptional control of liver regeneration. FASEB J. 1996;10(4):413–27. 8647340

[12] Fielding CA, Jones GW, McLoughlin RM, McLeod L, Hammond VJ, Uceda J, et al. Interleukin-6 Signaling Drives Fibrosis in Unresolved Inflammation. Immunity. 2014;40(1):40–50. 24412616 10.1016/j.immuni.2013.10.022 PMC3919204

[13] Maspero M, Yilmaz S, Cazzaniga B, Raj R, Ali K, Mazzaferro V, et al. The role of ischaemia-reperfusion injury and liver regeneration in hepatic tumour recurrence. Jhep Rep. 2023;5(11):100846. 10.1016/j.jhepr.2023.100846 PMC10523008 37771368

[14] Michalopoulos GK, DeFrances MC. Liver regeneration. Science. 1997;276(5309):60–6. 9082986 10.1126/science.276.5309.60

[15] Komposch K, Sibilia M. EGFR signaling in liver diseases. Int J Mol Sci 2016;17(1):30 . 10.3390/ijms17010030 PMC4730276 26729094

[16] Natarajan A, Wagner B, Sibilia M. The EGF receptor is required for efficient liver regeneration. Proc Natl Acad Sci U S A. 2007;104(43):17081–6. 17940036 10.1073/pnas.0704126104 PMC2040457

[17] Zunke F, Rose-John S. The shedding protease ADAM17: physiology and pathophysiology. Biochimica et Biophysica Acta (BBA). 2017;1864(11 Pt B):2059–70. 10.1016/j.bbamcr.2017.07.001 28705384

[18] Peschon JJ, Slack JL, Reddy P, Stocking KL, Sunnarborg SW, Lee DC, et al. An essential role for ectodomain shedding in mammalian development. Science. 1998;282(5392):1281–4. 9812885 10.1126/science.282.5392.1281

[19] Al-Salihi M, Bornikoel A, Zhuang Y, Stachura P, Scheller J, Lang KS, et al. The role of ADAM17 during liver damage. Biol Chem. 2021;402(9):1115–28. 34192832 10.1515/hsz-2021-0149

[20] Weiss TS, Lupke M, Dayoub R, Geissler EK, Schlitt HJ, Melter M, et al. Augmenter of liver regeneration reduces ischemia reperfusion injury by less chemokine expression, Gr-1 infiltration and oxidative stress. Cells. 2019;8(11):4121. 10.3390/cells8111421 PMC6912457 31718093

[21] Weiss TS, Lupke M, Ibrahim S, Buechler C, Lorenz J, Ruemmele P, et al. Attenuated lipotoxicity and apoptosis is linked to exogenous and endogenous augmenter of liver regeneration by different pathways. PLoS ONE. 2017;12(9):e0184282. 28877220 10.1371/journal.pone.0184282 PMC5587239

[22] Wang G, Yang X, Zhang Y, Wang Q, Chen H, Wei H, et al. Identification and characterization of receptor for mammalian hepatopoietin that is homologous to yeast ERV1. J Biol Chem. 1999;274(17):11469–72. 10206950 10.1074/jbc.274.17.11469

[23] Ohtsu H, Dempsey PJ, Eguchi S. ADAMs as mediators of EGF receptor transactivation by G protein-coupled receptors. Am J Physiol Cell Physiol. 2006;291(1):C1-10. 16769815 10.1152/ajpcell.00620.2005

[24] Schmitz AL, Schrage R, Gaffal E, Charpentier TH, Wiest J, Hiltensperger G, et al. A cell-permeable inhibitor to trap Gα proteins in the empty pocket conformation. Chem Biol. 2014;21(7):890–902. 25036778 10.1016/j.chembiol.2014.06.003 PMC4337399

[25] Xu PL, Liu JM, Sakaki-Yumoto M, Derynck R. TACE Activation by MAPK-Mediated regulation of cell surface dimerization and TIMP3 Association. Science Signaling. 2012;5(222) :ra34. 10.1126/scisignal.2002689 PMC4254802 22550340

[26] Poggi M, Kara I, Brunel JM, Landrier JF, Govers R, Bonardo B, et al. Palmitoylation of TNF alpha is involved in the regulation of TNF receptor 1 signalling. Biochim Biophys Acta. 2013;1833(3):602–12. 23159491 10.1016/j.bbamcr.2012.11.009

[27] Morris R, Kershaw NJ, Babon JJ. The molecular details of cytokine signaling via the JAK/STAT pathway. Protein Sci. 2018;27(12):1984–2009. 30267440 10.1002/pro.3519 PMC6237706

[28] Rose-John S. IL-6 trans-signaling via the soluble IL-6 receptor: importance for the pro-inflammatory activities of IL-6. Int J Biol Sci. 2012;8(9):1237–47. 23136552 10.7150/ijbs.4989 PMC3491447

[29] Gao B, Wang H, Lafdil F, Feng D. STAT proteins - key regulators of anti-viral responses, inflammation, and tumorigenesis in the liver. J Hepatol. 2012;57(2):430–41. 22504331 10.1016/j.jhep.2012.01.029 PMC3399024

[30] Horiguchi N, Wang L, Mukhopadhyay P, Park O, Jeong WI, Lafdil F, et al. Cell type-dependent pro- and anti-inflammatory role of signal transducer and activator of transcription 3 in alcoholic liver injury. Gastroenterology. 2008;134(4):1148–58. 18395093 10.1053/j.gastro.2008.01.016 PMC2376046

[31] Murthy A, Defamie V, Smookler DS, Di Grappa MA, Horiuchi K, Federici M, et al. Ectodomain shedding of EGFR ligands and TNFR1 dictates hepatocyte apoptosis during fulminant hepatitis in mice. J Clin Investig. 2010;120(8):2731–44. 20628198 10.1172/JCI42686 PMC2913323

[32] Wang WJ, Qiao YH, Li ZJ. New insights into modes of GPCR activation. Trends Pharmacol Sci. 2018;39(4):367–86. 29395118 10.1016/j.tips.2018.01.001

[33] Liebmann C. EGF receptor activation by GPCRs: an universal pathway reveals different versions. Mol Cell Endocrinol. 2011;331(2):222–31. 20398727 10.1016/j.mce.2010.04.008

[34] Harding SD, Sharman JL, Faccenda E, Southan C, Pawson AJ, Ireland S, et al. The IUPHAR/BPS Guide to PHARMACOLOGY in 2018: updates and expansion to encompass the new guide to IMMUNOPHARMACOLOGY. Nucleic Acids Res. 2018;46(D1):D1091–106. 29149325 10.1093/nar/gkx1121 PMC5753190

[35] Rozengurt E. Mitogenic signaling pathways induced by G protein-coupled receptors. J Cell Physiol. 2007;213(3):589–602. 17786953 10.1002/jcp.21246

[36] Gandhi CR. Augmenter of liver regeneration. Fibrogenesis Tissue Repair. 2012;5(1):10. 22776437 10.1186/1755-1536-5-10 PMC3519801

[37] Gandhi CR, Murase N, Starzl TE. Cholera toxin-sensitive GTP-binding protein-coupled activation of augmenter of liver regeneration (ALR) receptor and its function in rat kupffer cells. J Cell Physiol. 2010;222(2):365–73. 19859909 10.1002/jcp.21957 PMC3034370

[38] Palanisamy S, Xue C, Ishiyama S, Prasad SVN, Gabrielson K. GPCR-ErbB transactivation pathways and clinical implications. Cellular Signalling. 2021;86:11092. 10.1016/j.cellsig.2021.110092 34303814

[39] Schumacher N, Rose-John S. ADAM17 orchestrates Interleukin-6, TNFalpha and EGF-R signaling in inflammation and cancer. Biochim Biophys Acta. 2022;1869(1):119141. 10.1016/j.bbamcr.2021.119141 34610348

[40] Amour A, Slocombe PM, Webster A, Butler M, Knight CG, Smith BJ, et al. TNF-α converting enzyme (TACE) is inhibited by TIMP-3. FEBS Lett. 1998;435(1):39–44. 9755855 10.1016/s0014-5793(98)01031-x

[41] Ibrahim S, Dayoub R, Saberi V, Buchner M, Melter M, Weiss TS. Augmenter of Liver Regeneration (ALR) regulates bile acid synthesis and attenuates bile acid-induced apoptosis via glycogen synthase kinase-3β (GSK-3β) inhibition. Exp Cell Res. 2020;397(1):112343. 33132196 10.1016/j.yexcr.2020.112343

[42] Wang N, Wang Z, Sun H, Shi X, Zhang Y, Liu Q. Augmenter of liver regeneration improves therapeutic effect of hepatocyte homotransplantation in acute liver failure rats. Int Immunopharmacol. 2013;15(2):325–32. 23337881 10.1016/j.intimp.2013.01.002

[43] Long FW, Liang SY, Liu ZJ, Chen Y, Tu ZD, Shi YJ, et al. Augmentor of liver regeneration ameliorates renal tubular epithelial cell injury after rat liver transplantation. Transplant Proc. 2008;40(8):2696–9. 18929838 10.1016/j.transproceed.2008.08.015

[44] Papa S, Bubici C, Zazzeroni F, Franzoso G. Mechanisms of liver disease: cross-talk between the NF-kappaB and JNK pathways. Biol Chem. 2009;390(10):965–76. 19642868 10.1515/BC.2009.111 PMC2775491

[45] Kim H, Baumann H. Dual signaling role of the protein tyrosine phosphatase SHP-2 in regulating expression of acute-phase plasma proteins by interleukin-6 cytokine receptors in hepatic cells. Mol Cell Biol. 1999;19(8):5326–38. 10409724 10.1128/mcb.19.8.5326 PMC84376

[46] Wang Y, Ripperger J, Fey GH, Samols D, Kordula T, Wetzler M, et al. Modulation of hepatic acute phase gene expression by epidermal growth factor and Src protein tyrosine kinases in murine and human hepatic cells. Hepatology. 1999;30(3):682–97. 10462375 10.1002/hep.510300318

[47] Stahl N, Boulton TG, Farruggella T, Ip NY, Davis S, Witthuhn BA, et al. Association and activation of Jak-Tyk kinases by Cntf-Lif-Osm-Il-6 beta-receptor components. Science. 1994;263(5143):92–5. 8272873 10.1126/science.8272873

[48] Lokau J, Agthe M, Flynn CM, Garbers C. Proteolytic control of Interleukin-11 and Interleukin-6 biology. Bba-Mol Cell Res. 2017;1864(11):2105–17. 10.1016/j.bbamcr.2017.06.008 28630024

[49] Horiguchi N, Lafdil F, Miller AM, Park O, Wang H, Rajesh M, et al. Dissociation between liver inflammation and hepatocellular damage induced by carbon tetrachloride in myeloid cell-specific signal transducer and activator of transcription 3 gene knockout mice. Hepatology. 2010;51(5):1724–34. 20196117 10.1002/hep.23532 PMC2862139

[50] Mahmoud HM, Abouzed DEE, Abo-youssef AM, Hemeida RAM. Zafirlukast protects against hepatic ischemia-reperfusion injury in rats via modulating Bcl-2/Bax and NF-κB/SMAD-4 pathways. Intern Immunopharmacol 2023;122:110498. 10.1016/j.intimp.2023.110498 37418987

[51] Hafez T, Moussa M, Nesim I, Baligh N, Davidson B, Abdul-Hadi A. The effect of intraportal prostaglandin E1 on adhesion molecule expression, inflammatory modulator function, and histology in canine hepatic ischemia/reperfusion injury. J Surg Res. 2007;138(1):88–99. 17174338 10.1016/j.jss.2006.05.009

[52] Xu F, Liu XL, Wang C, Dai CL. Prostaglandin E1 preconditioning attenuates liver ischemia reperfusion injury in a rat model of extrahepatic cholestasis. Biomed Res Intern 2018;2018: 381242438. 10.1155/2018/3812424 PMC5817361 29511679

