# Supplementary material for: ADAM17, induced by Augmenter of Liver Regeneration via G protein-coupled receptor activation, transactivates epidermal growth factor-receptor and reduces classical IL-6 signaling
Source: Cell Commun Signal. 2026 Mar 7;24:214. doi: 10.1186/s12964-026-02782-7 (PMC13063610; doi:10.1186/s12964-026-02782-7)
Supplement: Supplementary file 1 — Supplementary Material 1. Supplementary methods. [file 12964_2026_2782_MOESM1_ESM.docx]

**Supplementary Methods:**

*IRI mouse model*

Liver samples from an ischaemia-reperfusion injury (IRI) mouse model were obtained from a previous study (1). Briefly, male wild-type (wt) C57BL/6 (B6) mice aged 6–8 weeks (Charles River Laboratories, Sulzfeld, Germany) weighing 19-22 g were used. Partial liver IRI was induced by placing an atraumatic clip across the portal vein, hepatic artery and bile duct just above branching to the right lateral lobe. This deprived 70% of the liver of blood flow. After 1.5 hours of ischaemia, the clamp was removed and the liver was reperfused for 3 hours. Sham-operated animals underwent the same procedure without clamping. The animals received intraperitoneal (i.p.) injections of recombinant human ALR (100 µg/kg body weight in 200 µl phosphate-buffered saline (PBS)) or PBS one hour before ischaemia and immediately before reperfusion. Liver tissue samples were collected from anaesthetized animals at the end of the ischaemic period.

*Primary mouse hepatocytes*

Primary mouse hepatocytes were isolated from wild-type C57BL/6 (B6) mice using the gentleMACS Octo Dissociator with Heaters and the Liver Perfusion Kit (#130-128-030, Miltenyi Biotec, Bergisch Gladbach, Germany) in accordance with the manufacturer's instructions. In summary, the left lateral lobe was meticulously dissected, thoroughly washed with phosphate-buffered saline (PBS), and then mounted in the designated perfuser. Following perfusion, the hepatocytes were subjected to enzymatic digestion and dissociation using the provided digestion buffers and programs. Thereafter, purification of the hepatocytes was conducted by filtration with 100 µm MACS SmartStrainers (Miltenyi Biotec, Bergisch Gladbach, Germany) and centrifugation (30 g, 5 min, 4 °C). Pelleted hepatocytes were resuspended in Dulbecco's Modified Eagle Medium (DMEM) (obtained from Thermo Fisher, Darmstadt, Germany), which was supplemented with penicillin (100 U/ml), streptomycin (100 µg/ml), and 5% fetal calf serum (purchased from Sigma-Aldrich, Taufkirchen, Germany). The viability of the isolated hepatocytes was determined using trypan blue exclusion, and cells with a viability of over 85% were used for cell culture. The cells were seeded onto collagen-coated culture dishes (Corning BioCoat, obtained from Fisher Scientific, Schwerte, Germany) at a density of 5 x 10^4^ cells/cm^2^. Following cell attachment, the medium was switched to serum-free medium for a period of 18-24 hours. Thereafter, the cells were treated according to the prescribed protocol. The viability of the hepatocytes during the culture period was monitored by cell morphology (light microscopy).

*Primary human hepatocytes*

Non-neoplastic human liver tissue for cell isolation was obtained from liver resections performed on patients undergoing partial hepatectomy for metastatic colorectal cancer liver tumors. Primary human hepatocytes (PHH) were isolated using the method described previously (2, 3). In brief, the cells were isolated using a modified two-step EGTA/collagenase perfusion procedure. The viability of the isolated hepatocytes was determined using trypan blue exclusion, and cells with a viability of over 85% were used for cell culture. The cells were plated at a density of 1.5 x 10⁵ cells/cm² in an appropriate volume of culture medium in collagen-coated 6-well plates and maintained at 37 °C in a humidified incubator with 5% CO₂. The medium consisted of DMEM supplemented with 5% fetal calf serum (FCS), 2 mM L-glutamine, and the following: 1.7 mU/ml insulin, 3.75 ng/ml hydrocortisone, 100 µg/ml streptomycin and 100 U/ml penicillin. After 16 hours, the medium was replaced with serum-free medium. The viability of the hepatocytes during the culture period was monitored by cell morphology (light microscopy) and by determination of the release of enzymes into the culture medium (aspartate aminotransferase activity).

References:

1. Weiss TS, Lupke M, Dayoub R, Geissler EK, Schlitt HJ, Melter M, et al. Augmenter of Liver Regeneration Reduces Ischemia Reperfusion Injury by Less Chemokine Expression, Gr-1 Infiltration and Oxidative Stress. Cells. 2019;8(11).

2. Damm G, Pfeiffer E, Burkhardt B, Vermehren J, Nussler AK, Weiss TS. Human parenchymal and non-parenchymal liver cell isolation, culture and characterization. Hepatol Int. 2013;7(4):951–8.

3. Weiss TS, Dayoub R. Thy-1 (CD90)-Positive Hepatic Progenitor Cells, Hepatoctyes, and Non-parenchymal Liver Cells Isolated from Human Livers. Methods Mol Biol. 2017;1506:75–89.
