# Supplementary material for: ADAM17, induced by Augmenter of Liver Regeneration via G protein-coupled receptor activation, transactivates epidermal growth factor-receptor and reduces classical IL-6 signaling
Source: Cell Commun Signal. 2026 Mar 7;24:214. doi: 10.1186/s12964-026-02782-7 (PMC13063610; doi:10.1186/s12964-026-02782-7)
Supplement: Supplementary file 2 — Supplementary Material 2. Table S1: Primary and secondary antibodies used for Western blotting. Table S2: Primers used in qRT-PCR experiments. Table S3: Densitometric analysis of western blots. [file 12964_2026_2782_MOESM2_ESM.pdf]

Table S1: Primary and secondary antibodies used for Western blotting.

Antibody	Provider	Catalog #
anti-ADAM17	Sigma-Aldrich, Taufkirchen, Germany	ABT94
anti-EGF-R (clone 528)	Proteintech, Martinsried, Germany	66455
anti-E-Cadherin	Abcam, Cambridge, UK	ab1416
anti-Akt	Cell Signaling, Frankfurt, Germany	2920
anti-p-Akt Ser473	Cell Signaling, Frankfurt, Germany	9271
anti-Erk1/2	Cell Signaling, Frankfurt, Germany	9102
anti-p-Erk1/2 Thr202/Tyr204	Cell Signaling, Frankfurt, Germany	4370
anti-Gα_q	Abcam, Cambridge, UK	ab128060
anti-gp130	Cell Signaling, Frankfurt, Germany	3732
anti-p-gp130 Ser782	Santa Cruz, Heidelberg, Germany	sc-377572
anti-JAK1	Cell Signaling, Frankfurt, Germany	3344
anti-p-JAK1 Tyr1034/1035	Cell Signaling, Frankfurt, Germany	3331
anti-JAK2	Cell Signaling, Frankfurt, Germany	3230
anti-p-JAK2 Tyr1007/1008	Cell Signaling, Frankfurt, Germany	3776
anti-SHP1	Cell Signaling, Frankfurt, Germany	3759
anti-p-SHP1 Tyr564	Cell Signaling, Frankfurt, Germany	8849
anti-SHP2	Abcam, Cambridge, UK	ab32083
anti-p-SHP2 Tyr542	Abcam, Cambridge, UK	ab62322
anti-Src	Cell Signaling, Frankfurt, Germany	2110
anti-p-Src Tyr416	Cell Signaling, Frankfurt, Germany	2101
anti-STAT3	Cell Signaling, Frankfurt, Germany	9139
anti-p-STAT3 Tyr705	Abcam, Cambridge, UK	ab76315
anti-TIMP3	Abcam, Cambridge, UK	ab85926
anti-p-Tyrosine	Cell Signaling, Frankfurt, Germany	96215
anti-beta-Actin	Cell Signaling, Frankfurt, Germany	4970
anti-GAPDH	Cell Signaling, Frankfurt, Germany	5174
HRP-conjugated anti-rabbit	Dako, Hamburg, Germany	P0448
HRP-conjugated anti-mouse	Dako, Hamburg, Germany	P0447

Table S2: Primers used in qRT-PCR experiments

Gene	Accession Nr.	Primer sequence (5' - 3')	
human ADAM10	NM_001110	*Fwd.*	CTGCCCAGCATCTGACCCTAA
		Rev.	TTGCCATCAGAACTGGCACAC
human ADAM17	NM_003183	*Fwd.*	GGTTCCTTTCGTGCTGGCGC
		Rev.	AAGCTTCTCGAGTCTCTGGTGGG
human FGB	NM_005141.5	*Fwd.*	CAGGGATTTGGAAATGTTGC
		Rev.	CCATCCTGGTAAGCTGGCTA
Human GNAQ	NM_002072.5	*Fwd.*	ACCGAATGGAGGAAAGCAAGG
		Rev.	CATCTCTCTGGGGTCCATCATATTC
human HAMP	NM_021175	*Fwd.*	AACCTACCTGCCCTGCCC
		Rev.	TTTGGAAAACAAAAGAACCAGCC
human HP	NM_005143.5	*Fwd.*	GCTAAGATGGTTTCCCACCA
		Rev.	TTTCGCTGTTGCATTTTCTG
human HPRT1	NM_000194	*Fwd.*	TGACACTGGCAAAACAATGCA
		Rev.	GGTCCTTTTCACCAGCAAGCT
human ICAM-1	NM_000201	*Fwd.*	GCTCAAGTGTCTAAAGGATGG
		Rev.	TATGACTGCGGCTGCTAC
human mgp80	NM_000565	*Fwd.*	CATTGCCATTGTTCTGAGGTTC
		Rev.	GTGCCACCCAGCCAGCTATC
human mgp130	NM_002184	*Fwd.*	AACACATCTGGCCTAATGTTCC
		Rev.	TACCACTGCTGTGTCCTTCAGT
human SAA2	NM_030754.5	*Fwd.*	AGCCAATTACATCGGCTCAG
		Rev.	ATTTATTGGCAGCCTGATCG
human sgp130	NM_002184	*Fwd.*	GCAGCATACACAGATGAAGGTG
		Rev.	TAAGCTGTAAGGTCCTCGTTGG
human TIMP3	NM_000362	*Fwd.*	CCAGGACGCCTTCTGCAA
		Rev.	GAAGAAGCTGGTAAAGGAGGGG
human total gp130	NM_002184	*Fwd.*	GAACAGCATCCAGTGTCACC
		Rev.	CATTTTCTTCCCCTCGTTCAC
mouse HAMP	NM_032541	*Fwd.*	CAGCACCACCTATCTCCATCAAC
		Rev.	CAGATGGGGAAGTTGGTGTCTC
mouse ICAM-1	NM_010493	*Fwd.*	CAATTTCTCATGCCGCACAG
		Rev.	AGCTGGAAGATCGAAAGTCCG

Table S3: Densitometric analysis of western blots. Protein expression was quantified densitometrically, with values given relative to the protein levels in the gray-shaded column. These levels were set as an expression of 1. The densitometric results of the cell experiments depicted in the manuscript are presented in bold. The number of independent biological replicates (n), summary statistics (mean ± SD), and statistical testing, when applicable, are provided.

**Fig. 1A**		-	α-EGF-R	-	α-EGF-R	-	α-EGF-R
	(n = 1)	-	-	ALR	ALR	EGF	EGF
p-Tyr/EGFR	**Hep3B**	0.16	0.22	0.33	0.05	1.00	0.13

**Fig. 1B**		-	α-EGF-R	-	α-EGF-R	-	α-EGF-R
	(n = 3)	-	-	ALR	ALR	EGF	EGF
p-Erk/Erk	**Hep3B**	0.24	0.10	0.55	0.07	1.00	0.14
	Huh7	0.18	0.17	0.38	0.16	1.00	0.38
	Huh7	0.35	0.17	0.67	0.25	1.00	0.39
	mean ± SD	*0.26 ± 0.07*	*0.15 ± 0.03*	*0.53 ± 0.12 **	*0.16 ± 0.07*	*1.00*	
	(n = 3)						
p-Akt/Akt	**Hep3B**	0.23	0.10	0.33	0.02	1.00	0.05
	Hep3B	0.08	0.11	0.22	0.01	1.00	0.02
	Huh7	0.08	0.08	0.26	0.14	1.00	0.05
	mean ± SD	*0.13 ± 0.07*	*0.10 ± 0.01*	*0.27 ± 0.05 **	*0.06 ± 0.06*	*1.00*	

* p<0.05 different from corresponding untreated treated cells.

**Fig. 1C/1B/S1B**		-	α-EGF-R	AG1478	-	α-EGF-R	AG1478	-	α-EGF-R	AG1478
	(n = 6)	-	-	-	EGF	EGF	EGF	ALR	ALR	ALR
p-Erk/Erk	**Huh7** (Fig. 1C)	0.01	0.00	0.10	1.00	0.06	0.32	0.55	0.01	0.12
	**HepG2** (Fig. 1C)	0.50	0.39	0.58	1.00	0.36	0.51	1.51	0.69	0.88
	**Hep3B** (Fig. 1B)	0.24	0.10		1.00	0.14		0.55	0.07	
	**Hep3B** (Fig. S1B)	0.10			1.00		0.10	0.29		0.06
	Huh7	0.01	0.04	0.06	1.00	0.22	0.04	0.38	0.08	0.01
	**Huh7** (Fig. S1B)	0.12			1.00		0.11	0.14		0.01
	mean ± SD	*0.17 ± 0.17*	*0.13 ± 0.15*	*0.24 ± 0.23*	*1.00*			*0.57 ± 0.44 **	*0.21 ± 0.28*	*0.22 ± 0.33*

* p<0.05 different from corresponding untreated treated cells.

**Fig. 2A/B**		-	-	ALR		EGF	
	(n = 3)	-	BIM46187	-	BIM46187	-	BIM46187
p-Erk/Erk	**Hep3B** (Fig. 2A)	0.03	0.04	**0.26**	**0.10**	**1.00**	**0.94**
	**Huh7** (Fig. 2B)	0.12	0.03	0.38	0.02	1.00	-
	**HepG2** (Fig. S1A)	0.01	0.01	**0.26**	**0.02**	**1.00**	**1.11**
	mean ± SD	*0.06 ± 0.05*	*0.03 ± 0.01*	*0.30 ± 0.06 **	*0.04 ± 0.04*	*1.00*	
	(n = 1)						
p-Akt/Akt	**Hep3B** (Fig. 2A)	0.06	0.08	**0.53**	**0.33**	**1.00**	**0.92**
	mean ± SD					*1.00*	
	(n = 1)						
p-Src/Src	**Huh7** (Fig 2B)	0.21	0.35	**1.14**	**0.60**	**1.00**	-

* p<0.05 different from corresponding untreated treated cells.

Fig. 2C/S1B

		-	-		ALR	
	(n = 4)	-	eCF506	EGF	-	eCF506
p-Erk/Erk	**Huh7**	0.07	0.05	1.00	0.38	0.01
	**Hep3B**	0.09	0.11	1.00	0.22	0.09
	**Hep3B** (Fig. S1B)	0.10		1.00	0.19	0.07
	**Huh7** (Fig. S1B)	0.12		1.00	0.14	0.04
	mean ± SD	*0.09 ± 0.02*	*0.08 ± 0.03*	*1.00*	*0.27 ± 0.10 **	*0.05 ± 0.03*

	(n = 2)					
p-Src/Src	**Huh7** (Fig. 2C)	0.19	0.04	**1.00**	**0.45**	**0.00**
	Huh7	0.45	0.03	1.00	1.26	0.01
	mean ± SD	*0.32 ± 0.13*		*1.00*	*0.85 ± 0.41*	*0.00 ± 0.00*

* p<0.05 different from untreated treated cells.

Fig. 3A

		-	-	GW	-	GW
	(n = 1)	-	ALR	ALR	EGF	EGF
p-Tyr/EGFR	**Hep3B**	0.21	0.52	0.21	1.00	0.89

Fig. 3B

		-	-	-	EGF			ALR					
	(n = 3)	-	GW	Marimastat	-	GW	Marimastat	-	GW	Marimastat	-	GW	GI
p-Erk/Erk	**Hep3B**	0.11	0.06	0.04	1.00	0.90	0.89	1.00	0.13	0.19	1.00	0.51	0.97
	Hep3B	0.12	0.10	0.06	1.00	-	-	1.00	0.21	0.30	1.00	0.71	1.02
	HepG2	0.07	0.11	0.21	1.00	-	-	1.00	0.71	0.46	1.00	0.61	1.03
	mean ± SD	*0.10 ± 0.02*						*1.00*	*0.35 ± 0.26 **	*0.32 ± 0.11 **	*1.00*	*0.61 ± 0.08 **	*1.01 ± 0.02*

	(n = 2)												
p-Akt/Akt	**Hep3B**	0.00	0.00	0.00	1.00	1.39	1.93	1.00	0.22	0.23	1.00	0.77	1.05
	HepG2	0.12	-	-	1.00	1.17	1.24	1.00	0.49	0.48	1.00	0.53	0.97
	mean ± SD	*0.06 ± 0.06*						*1.00*	*0.35 ± 0.14*	*0.36 ± 0.12*	*1.00*	*0.65 ± 0.12*	*1.01 ± 0.04*

* p<0.05 different from corresponding ALR treated cells.

Fig. 3C

	(n = 1)	-	-	-	eCF506			GW		
		-	ALR	EGF	-	ALR	EGF	-	ALR	EGF
p-Akt/Akt	**PMH**	0.07	0.11	1.00	0.00	0.00	0.39	0.03	0.02	0.77

Fig 3D

	(n = 1)	-	-	-	sc siRNA			GNAQ siRNA			ADAM17 siRNA		
		-	ALR	EGF	-	ALR	EGF	-	ALR	EGF	-	ALR	EGF
p-Erk/Erk	**Hep3B**	0.31	0.44	1.00	**0.33**	**0.47**	**1.00**	**0.33**	**0.34**	**1.00**	**0.18**	**0.20**	**1.00**
	(n = 1)												
p-Akt/Akt	**Hep3B**	0.15	0.44	1.00	**0.25**	**0.46**	**1.00**	**0.34**	**0.32**	**1.00**	**0.14**	**0.16**	**1.00**

Fig. 3F

	(n = 4)	Cytosol			Membrane		
		-	ALR	PMA	-	ALR	PMA
mature/pro ADAM17	**HepG2**	-	-	-	**1.00**	**1.46**	1.03
	HepG2	-	-	-	1.00	1.22	1.24
	**Huh7**	-	-	-	**1.00**	**1.81**	1.19
	Huh7	-	-	-	1.00	1.35	0.87
	mean ± SD				*1.00*	*1.46 ± 0.22 **	*1.08 ± 0.15*
total ADAM17/E-Cadherin	**HepG2**	-	-	-	**1.00**	**2.19**	**1.38**
	HepG2	-	-	-	1.00	1.31	0.47
	**Huh7**	-	-	-	**1.00**	**1.42**	**1.50**
	Huh7	-	-	-	1.00	1.23	1.29
	mean ± SD				*1.00*	*1.54 ± 0.39*	*1.16 ± 0.40*

* p<0.05 different from untreated treated cells.

Fig. 3G

	(n = 1)	-	-	-	-	EGF				ALR			
		-	α-TGFα	α-AREG	α-HB-EGF	-	α-TGFα	α-AREG	α-HB-EGF	-	α-TGFα	α-AREG	α-HB-EGF
p-Erk/Erk	**HepG2**	0.18	0.22	0.22	0.27	1.00	1.01	1.66	1.60	1.00	0.48	0.43	0.95

Fig. 4A

	(n = 3)	-	IL-6	
		-	-	ALR
p-STAT3/ STAT3	**PMH,** M1	0.57	**1.00**	0.28
	**PMH,** M2	0.35	**1.00**	0.32
	**PMH,** M3	0.33	**1.00**	0.52
	mean ± SD	*0.42 ± 0.11*	*1.00*	*0.37 ± 0.11*

* p<0.05 different from IL-6 treated cells.

Fig. 4B

	(n = 2)	-	-	IL-6		IL-6		IL-6	
		-	ALR	-	ALR	AG1478	AG1478 + ALR	α-EGF-R	α-EGF-R + ALR
p-STAT3/ STAT3	**Hep3B**	0.00	0.01	**1.00**	**0.60**	**1.00**	**0.46**	**1.00**	**0.67**
	Hep3B	0.00	0.00	1.00	0.64	1.00	0.84	1.00	0.86
	mean ± SD			*1.00*	*0.62 ± 0.02*	*1.00*	*0.65 ± 0.19*	*1.00*	*0.76 ± 0.09*

Fig. 4D

	(n = 2)	-	-	-	-	NSC-87877			
		-	ALR	IL-6	ALR + IL-6	-	ALR	IL-6	IL-6 + ALR
p-STAT3/ STAT3	**Huh7**	0.01	0.02	**1.00**	**0.75**	0.01	0.01	**1.00**	**0.84**
	Huh7	0.13	0.06	1.00	0.79	0.14	0.15	1.00	0.78
	mean ± SD			*1.00*	*0.77 ± 0.02*			*1.00*	*0.81 ± 0.03*

	(n = 3)	-	-	-	-	TPI-1			
		-	ALR	IL-6	ALR + IL-6	-	ALR	IL-6	IL-6 + ALR
p-STAT3/ STAT3	**HepG2**	0.04	0.00	**1.00**	**0.72**	0.03	0.00	**1.00**	**0.03**
	Huh7	0.12	0.06	1.00	0.73	0.09	0.07	1.00	0.51
	HepG2	0.00	0.00	1.00	0.77	0.00	0.01	1.00	0.58
	mean ± SD			*1.00*	*0.74 ± 0.02 **			*1.00*	*0.37 ± 0.25 **

	(n = 2)	sc. siRNA				SHP1 siRNA			
		-	ALR	IL-6	ALR + IL-6	-	ALR	IL-6	IL-6 + ALR
p-STAT3/ STAT3	**HepG2**	0.18	0.13	**1.00**	**0.73**	0.04	0.07	**1.00**	**0.53**
	HepG2	0.07	0.04	1.00	0.80	0.02	0.00	1.00	0.62
	mean ± SD			*1,00*	*0,77 ± 0.03*			*1,00*	*0,57 ± 0.05*

* p<0.05 different from corresponding IL-6 treated cells.

Fig. 5B

	(n = 3)	-	-	-	-	-	-	GW					
		-	ALR	PMA	IL-6	IL-6 + ALR	IL-6 + PMA	-	ALR	PMA	IL-6	IL-6 + ALR	IL-6 + PMA
p-STAT3/ STAT3	**Hep3B**	0.00	0.00	0.00	**1.00**	**0.47**	0.08	0.00	0.00	0.00	**1.00**	**0.94**	0.97
	**HepG2** (Fig. S6)	0.23	0.18	0.05	**1.00**	**0.72**	0.18	0.18	0.18	0.11	**1.00**	**1.02**	0.47
	**Huh7** (Fig. S6)	0.06	0.04	0.00	**1.00**	**0.63**	0.21	0.03	0.05	0.00	**1.00**	**1.05**	0.75
	mean ± SD				*1.00*	*0.61± 0.11 **					*1.00*	*1.00± 0.05*	

* p<0.05 different from corresponding IL-6 treated cells.

	(n = 3)	-	-	-	-	-	-	Marimastat					
		-	ALR	PMA	IL-6	IL-6 + ALR	IL-6 + PMA	-	ALR	PMA	IL-6	IL-6 + ALR	IL-6 + PMA
p-STAT3/ STAT3	**Hep3B**	0.05	0.04	0.03	**1.00**	**0.54**	0.13	0.01	0.04	0.03	**1.00**	**0.99**	1.04
	HepG2 (Fig. S6)	0.00	0.00	0.00	**1.00**	**0.35**	0.27	0.00	0.00	0.00	**1.00**	**0.96**	0.53
	Huh7 (Fig. S6)	0.00	0.00	0.00	**1.00**	**0.59**	0.12	0.00	0.01	0.00	**1.00**	**1.04**	0.40
	mean ± SD				*1.00*	*0.50 ± 0.10 **					*1.00*	*1.00 ± 0.03*	

* p<0.05 different from corresponding IL-6 treated cells.

Fig. 5C

	(n = 2)	-	-	-	-	-	-	GW			GI		
		-	ALR	PMA	IL-6	IL-6 + ALR	IL-6 + PMA	IL-6	IL-6 + ALR	IL-6 + PMA	IL-6	IL-6 + ALR	IL-6 + PMA
p-STAT3/ STAT3	**HepG2**	0.00	0.00	0.00	**1.00**	**0.25**	0.17	**1.00**	**0.97**	1.04	**1.00**	**0.65**	0.21
	HepG2	0.01	0.01	0.00	1.00	0.49	0.07	1.00	0.99	0.29	1.00	0.25	0.10
	mean ± SD				*1.00*	*0.37 ± 0.12*		*1.00*	*0.98 ± 0.01*		*1.00*	*0.45 ± 0.20*	

**Fig. 5D**		-	-	-	-	sc siRNA				ADAM17 siRNA			
	(n = 2)	-	IL-6	IL-6 + ALR	IL-6 + PMA	-	IL-6	IL-6 + ALR	IL-6 + PMA	-	IL-6	IL-6 + ALR	IL-6 + PMA
p-STAT3/ STAT3	**HepG2**	0.00	**1.00**	**0.82**	0.27	0.01	**1.00**	**0.77**	0.43	0.01	**1.00**	**0.95**	1.05
	HepG2	0.06	1.00	0.80	0.65	0.11	1.00	0.76	0.70	0.06	1.00	0.94	1.37
	mean ± SD		*1.00*	*0.81 ± 0.01*			*1.00*	*0.76 ± 0.01*			*1.00*	*0.95 ± 0.01*	

**Fig. 5E**		-	-	-	-	sc siRNA				GNAQ siRNA			
	(n = 2)	-	ALR	IL-6	IL-6 + ALR	-	ALR	IL-6	IL-6 + ALR	-	ALR	IL-6	IL-6 + ALR
p-STAT3/ STAT3	**HepG2**	0.02	0.00	**1.00**	**0.70**	0.03	0.02	**1.00**	**0.71**	0.03	0.02	**1.00**	**1.10**
	HepG2	0.03	0.01	1.00	0.81	0.01	0.02	1.00	0.65	0.06	0.06	1.00	0.92
	mean ± SD			*1.00*	*0.75 ± 0.05*			*1.00*	*0.68 ± 0.03*			*1.00*	*1.01 ± 0.09*

**Fig. 5F**		-	-	-	-	-	-	eCF506					
	(n = 3)	-	ALR	PMA	IL-6	IL-6 + ALR	IL-6 + PMA	-	ALR	PMA	IL-6	IL-6 + ALR	IL-6 + PMA
p-STAT3/ STAT3	**Hep3B**	0.00	0.01	0.00	**1.00**	**0.54**	0.44	0.00	0.01	0.00	**1.00**	**0.99**	0.71
	Huh7	0.34	0.34	0.40	1.00	0.65	0.64	0.14	0.14	0.06	1.00	0.99	0.82
	HepG2	0.01	0.00	0.00	1.00	0.73	0.13	0.01	0.01	0.00	1.00	0.95	0.20
	mean ± SD				*1.00*	*0.64 ± 0.08 **					*1.00*	*0.98 ± 0.02*	

* p<0.05 different from corresponding IL-6 treated cells.

**Fig. 5G**		-	-	-	-	-	-	BIMX					
	(n = 2)	-	ALR	PMA	IL-6	IL-6 + ALR	IL-6 + PMA	-	ALR	PMA	IL-6	IL-6 + ALR	IL-6 + PMA
p-STAT3/ STAT3	**HepG2**	0.00	0.00	0.00	**1.00**	**0.50**	0.39	0.01	0.01	0.00	**1.00**	**1.04**	1.00
	Hep3B	0.00	0.00	0.00	1.00	0.69	0.22	0.00	0.00	0.00	1.00	1.21	1.07
	mean ± SD				*1.00*	*0.60 ± 0.10*					*1.00*	*1.13 ± 0.08*	
