# Supplementary figures and images for: ADAM17, induced by Augmenter of Liver Regeneration via G protein-coupled receptor activation, transactivates epidermal growth factor-receptor and reduces classical IL-6 signaling
Source: Cell Commun Signal. 2026 Mar 7;24:214. doi: 10.1186/s12964-026-02782-7 (PMC13063610; doi:10.1186/s12964-026-02782-7)

**A**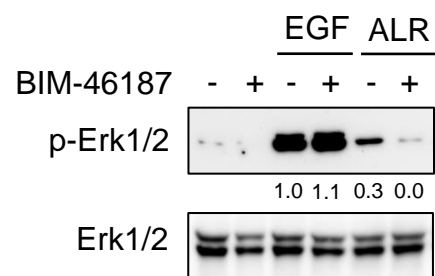**B**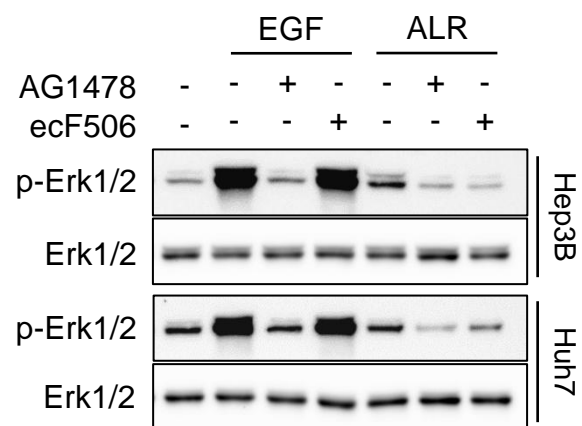

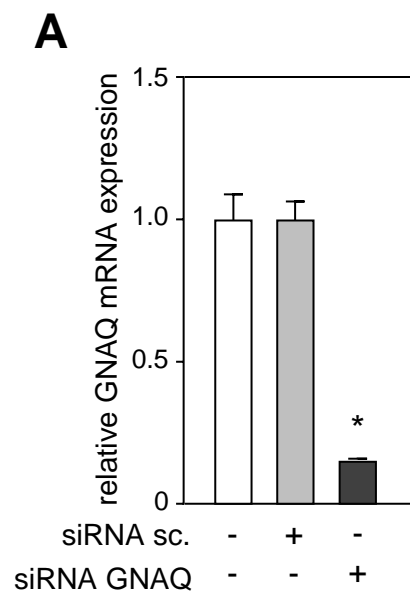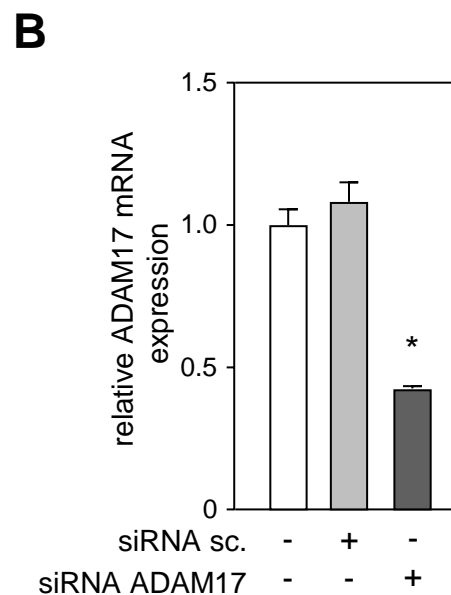

**A**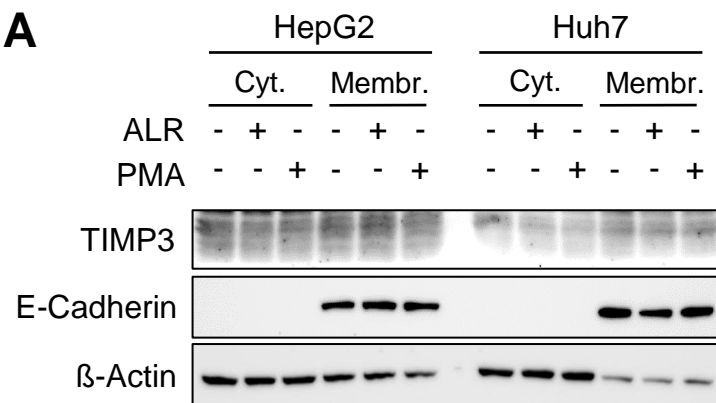**B**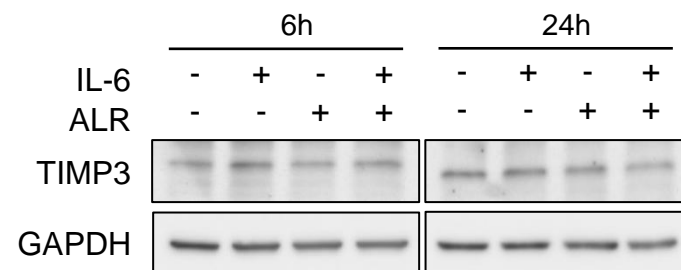

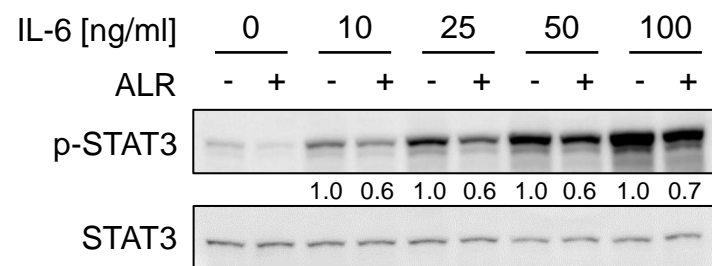

Fig. S4

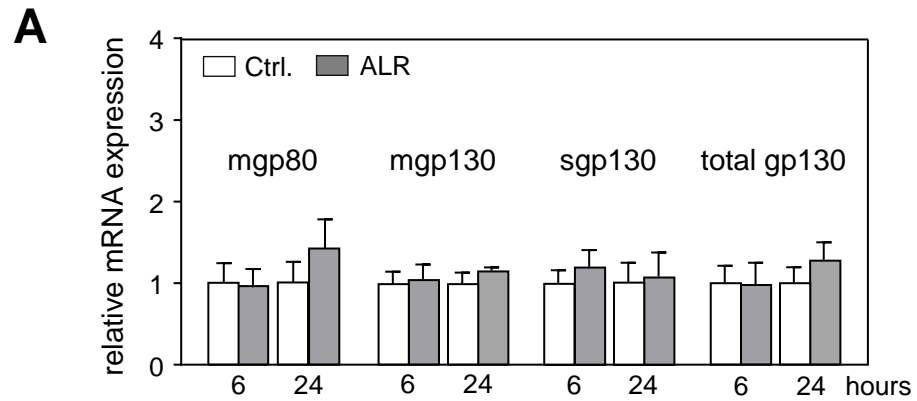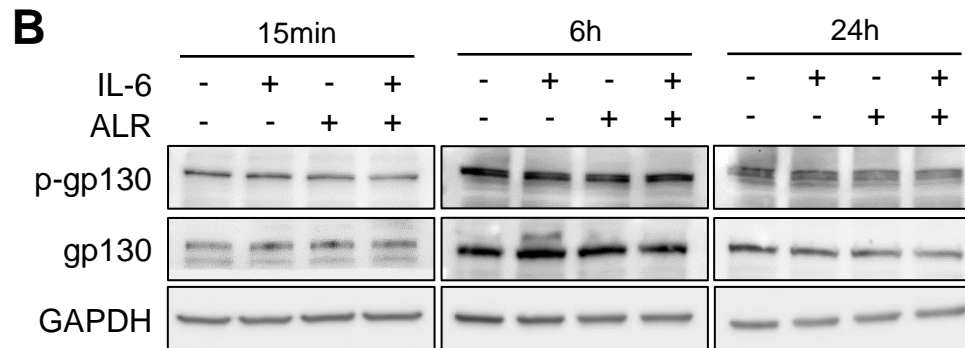

Fig. S5

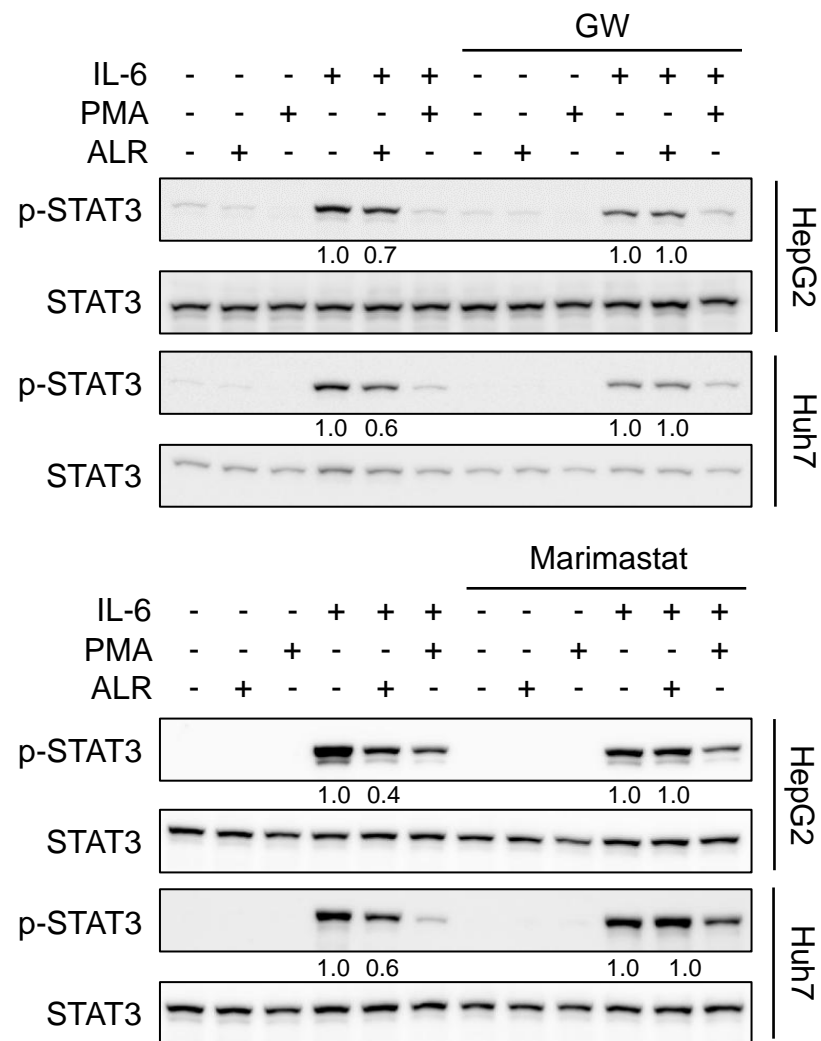

Fig. S6

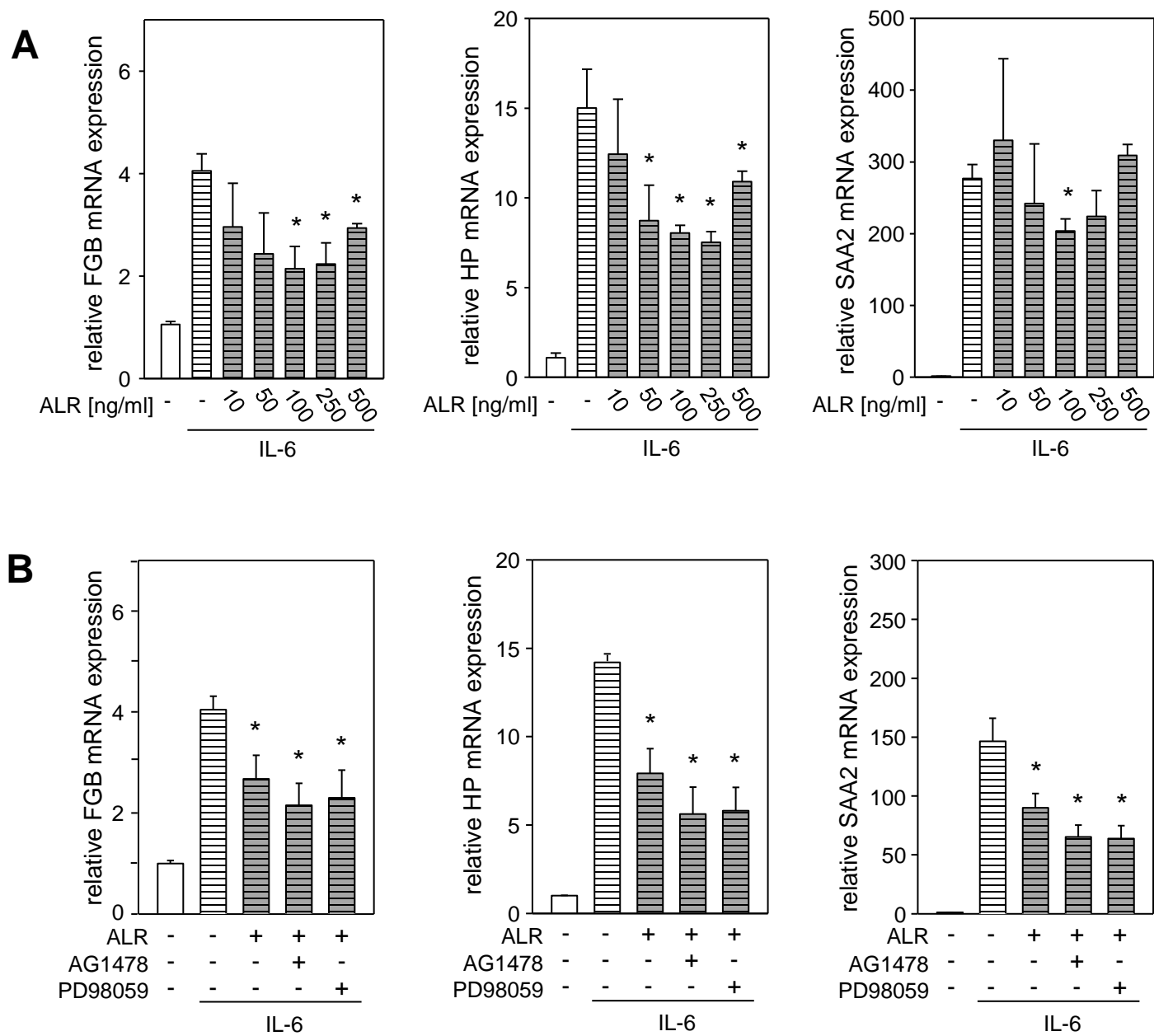

Fig. S7

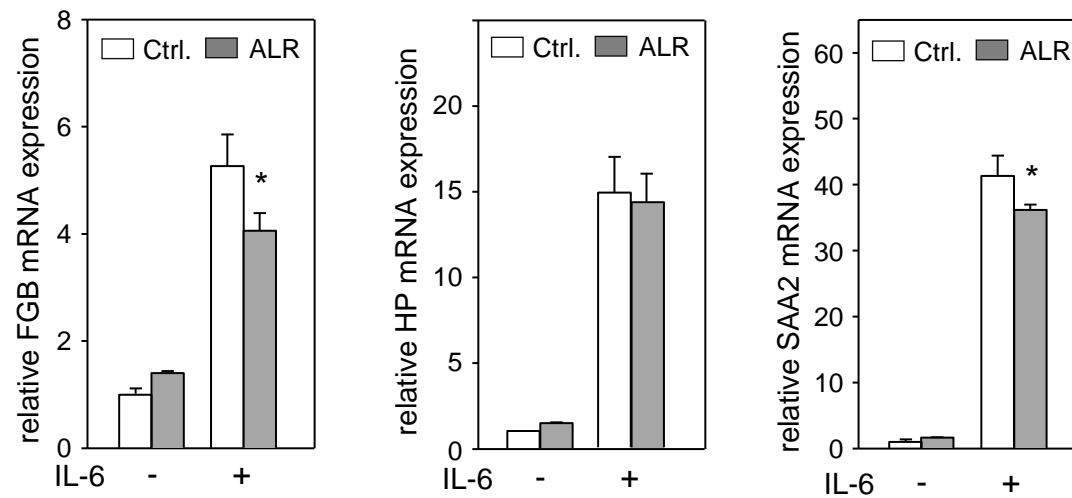

Fig. S8

# Graphical summary

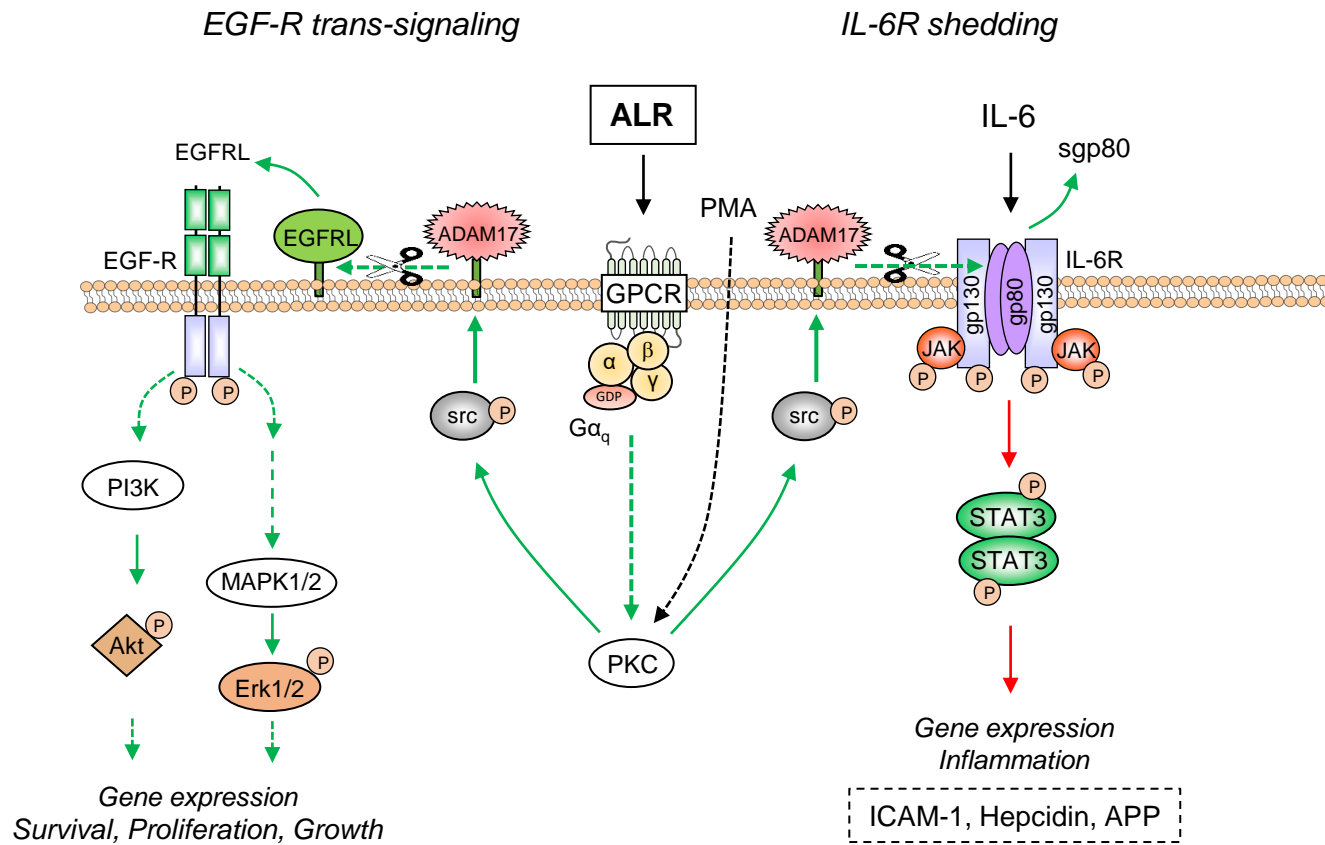

Fig. S9

Supplement: Supplementary file 3 — Supplementary Material 3. Figures S1 to S9. [file 12964_2026_2782_MOESM3_ESM.pdf]
