# Supplementary material for: ADAM17, induced by Augmenter of Liver Regeneration via G protein-coupled receptor activation, transactivates epidermal growth factor-receptor and reduces classical IL-6 signaling
Source: Cell Commun Signal. 2026 Mar 7;24:214. doi: 10.1186/s12964-026-02782-7 (PMC13063610; doi:10.1186/s12964-026-02782-7)
Supplement: Supplementary file 4 — Supplementary Material 4. Legends of Figure S1 to S9. Figure S1: ALR induces Erk1/2 phosphorylation dependent on GPCR and Src activation upstream of EGF-R. Figure S2: Silencing of GNAQ and ADAM17 mRNA expression. Figure S3: Expression of the ADAM17 inhibitor TIMP3 is not induced upon ALR treatment in vitro. Figure S4: ALR attenuates IL-6-induced STAT3 phosphorylation. Figure S5: Administration of ALR does not change mRNA expression of IL-6-receptor subunits αand βnor degradation of gp130 protein. Figure S6: ALR attenuates IL-6-induced STAT3 phosphorylation by activation of sheddase ADAM17 in hepatoma cell lines. Figure S7: ALR reduces IL-6 induced acute phase proteins in hepatoma cells. Figure S8: ALR reduces IL-6 induced acute phase proteins in primary human hepatocytes. Figure S9: Graphical summary. [file 12964_2026_2782_MOESM4_ESM.docx]

**Supplementary figures:**

**Figure S1**: *ALR induces Erk1/2 phosphorylation dependent on GPCR and Src activation upstream of EGF-R.* **A**) HepG2 cells, pre-incubated without or with GPCR inhibitor BIM46187 (5 µM, 30 min), were treated with EGF (10 ng/ml) or ALR (100 ng/ml) for 20 min and immunoblotted for Erk1/2 phosphorylation. **B**) Hep3B and Huh7 cells were pre-incubated without or with the EGF-R-tyrosine kinase inhibitor AG1478 (10 µM, 30 min) or Src inhibitor eCF506 (1 µM, 30 min). The cells were then treated with EGF (10 ng/ml) or ALR (100 ng/ml) for 20 min and immunoblotted for Erk1/2 phosphorylation. ALR treatment increased p-Erk1/2, which is blunted by EGF-R or Src inhibition, while EGF-induced p-Erk1/2 is only reduced by EGF-R inhibition. Information regarding replicates, densitometric and statistical analysis can be found in supplementary Table S3.

**Figure S2:** *Silencing of GNAQ (G_αq_) and ADAM17 mRNA expression.* HepG2 cells were treated as described in Material and Methods with specific siRNAs for GNAQ (G_αq_) and ADAM17 for 24 hours. Scrambled (sc.) siRNA was used as control. The mRNA expression levels of GNAQ and ADAM17 were analyzed through the utilization of qRT-PCR and subsequently normalized to the housekeeping gene HPRT1 (three independent experiments, mean ± SD). * *p* < 0.05 differs from corresponding scrambled control (sc) siRNA.

**Figure S3**: *Expression of the ADAM17 inhibitor TIMP3 is not induced upon ALR treatment in vitro.* **A**) HepG2 or Huh7 cells were treated with PMA (20 nM, 10 min) or ALR (100 ng/ml, 20 min). Separated cytosolic (Cyt.) and membrane (Membr.) cell fractions were immunoblotted for TIMP3 expression with E-cadherin and β-actin as loading controls. **B**) HepG2 cells, pre-incubated without or with ALR (100 ng/ml, 60 min), were treated with IL-6 (25 ng/ml) for 6 and 24 hours and immunoblotted for TIMP3 expression with GAPDH as loading control.

**Figure S4**: *ALR attenuates IL-6-induced STAT3 phosphorylation.* HepG2 cells were pre-incubated without or with ALR (100 ng/ml, 60 min) and subsequently were treated with increasing concentrations of IL-6 for 15 min. Densitometric analysis was then performed for STAT3 phosphorylation (ratio p-STAT3/STAT3) and normalized to the corresponding IL-6-treated cells.

**Figure S5**: *Administration of ALR does not change mRNA expression of IL-6-receptor subunits α (gp80) and β (gp130) nor degradation of gp130 protein.* **A**) HepG2 cells were exposed to ALR (100 ng/ml) for 6 or 24 hours and the mRNA levels of membrane-bound IL-6-R subunits α (mgp80) and β (mgp130), soluble gp130 (sgp130), and total gp130 were analyzed by qRT-PCR and normalized to HPRT1 (three independent experiments, mean ± SD). **B**) HepG2 cells, pre-incubated without or with ALR (100 ng/ml, 60 min), were treated with IL-6 (25 ng/ml) for indicated times and immunoblotted for gp130 phosphorylation with GAPDH as loading control.

**Figure S6**: *ALR attenuates IL-6-induced STAT3 phosphorylation by activation of sheddase ADAM17 in hepatoma cell lines*. HepG2 and Huh7 cells were pre-incubated without or with GW (GW280264X, 20 µM, 90 min), Marimastat (20 µM, 90 min), PMA (20 nM, 10 min) or ALR (100 ng/ml, 60 min). Thereafter, the cells were treated with IL-6 (25 ng/ml) for 15 min and immunoblotted for STAT3 phosphorylation. Densitometric analysis was performed for STAT3 phosphorylation (ratio p-STAT3/STAT3) and normalized to IL-6-treated cells without ALR or PMA incubation. Information regarding replicates, densitometric and statistical analysis can be found in supplementary Table S3.

**Figure S7**: *ALR reduces IL-6 induced acute phase proteins in hepatoma cells.* **A**) HepG2 cells were pre-incubated without or with increasing concentrations of ALR for 60 min, treated with IL-6 (25 ng/ml) for 24 hours, and the mRNA levels of fibrinogen β (FGB), haptoglobin (HP) and SAA2 were analyzed by qRT-PCR. **B**) HepG2 cells were pre-incubated without or with EGF-R tyrosine kinase inhibitor AG1478 (1 µM, 90 min) or Erk1/2 inhibitor PD98059 (10µM, 90 min), and ALR (100 ng/ml, 60 min). The cells were then treated with IL-6 (25 ng/ml) for 24 hours, after which the mRNA levels of fibrinogen β (FGB), haptoglobin (HP) and SAA2 were analyzed by qRT-PCR. Data have been normalized to HPRT1 (three independent experiments, mean ± SD). * p<0.05 different from corresponding IL-6 treated cells.

**Figure S8**: *ALR reduces IL-6 induced acute phase proteins in primary human hepatocytes (PHH).* PHH, pre-incubated without or with ALR (100 ng/ml) for 60 min, were treated with IL-6 (25 ng/ml) for 24 hours and mRNA levels of fibrinogen β (FGB), haptoglobin (HP) and SAA2 were analyzed by qRT-PCR. Data have been normalized to HPRT1 (three independent experiments, mean ± SD). * p<0.05 different from corresponding IL-6 treated cells.

**Figure S9**: *Graphical summary.* Activation of a G-protein-coupled receptor (GPCR) on hepatocytes by ALR subsequently activates protein kinase C (PKC) and Src via phosphorylation, thereby increasing activity of metalloproteinase ADAM17 on the plasma membrane. This leads to ectodomain shedding of membrane-bound EGF-R ligands (EGF-RL), such as HB-EGF, TGFα, and AREG. The binding of these ligands to the EGF-R results in EGF-R phosphorylation (EGF-R trans-signaling), which, in turn, leads to the activation of the Akt and MAPK (Erk1/2) pathways, thereby stimulating gene expression involved in proliferation as well as anti-apoptotic activity. In addition, the ADAM17 protein, activated by ALR, is shown to cleave and release gp80, a subunit of the IL-6 receptor (IL-6R). This results in reduced IL-6-induced signaling, as evidenced by diminished STAT3 phosphorylation. Consequently, ALR-reduced IL-6 signaling leads to a decrease in the expression of APPs, hepcidin and ICAM-1. In summary, ALR treatment supports liver regeneration by stimulating EGF-R signaling, leading to an anti-apoptotic reaction and alleviates IL-6 induced signaling, resulting in an anti-inflammatory reaction, as evidenced by low apoptosis and less neutrophil infiltration in ischemia reperfusion-injured livers.
