# Supplementary material for: ADAM17, induced by Augmenter of Liver Regeneration via G protein-coupled receptor activation, transactivates epidermal growth factor-receptor and reduces classical IL-6 signaling
Source: Cell Commun Signal. 2026 Mar 7;24:214. doi: 10.1186/s12964-026-02782-7 (PMC13063610; doi:10.1186/s12964-026-02782-7)

Fig. 1 uncropped blot images

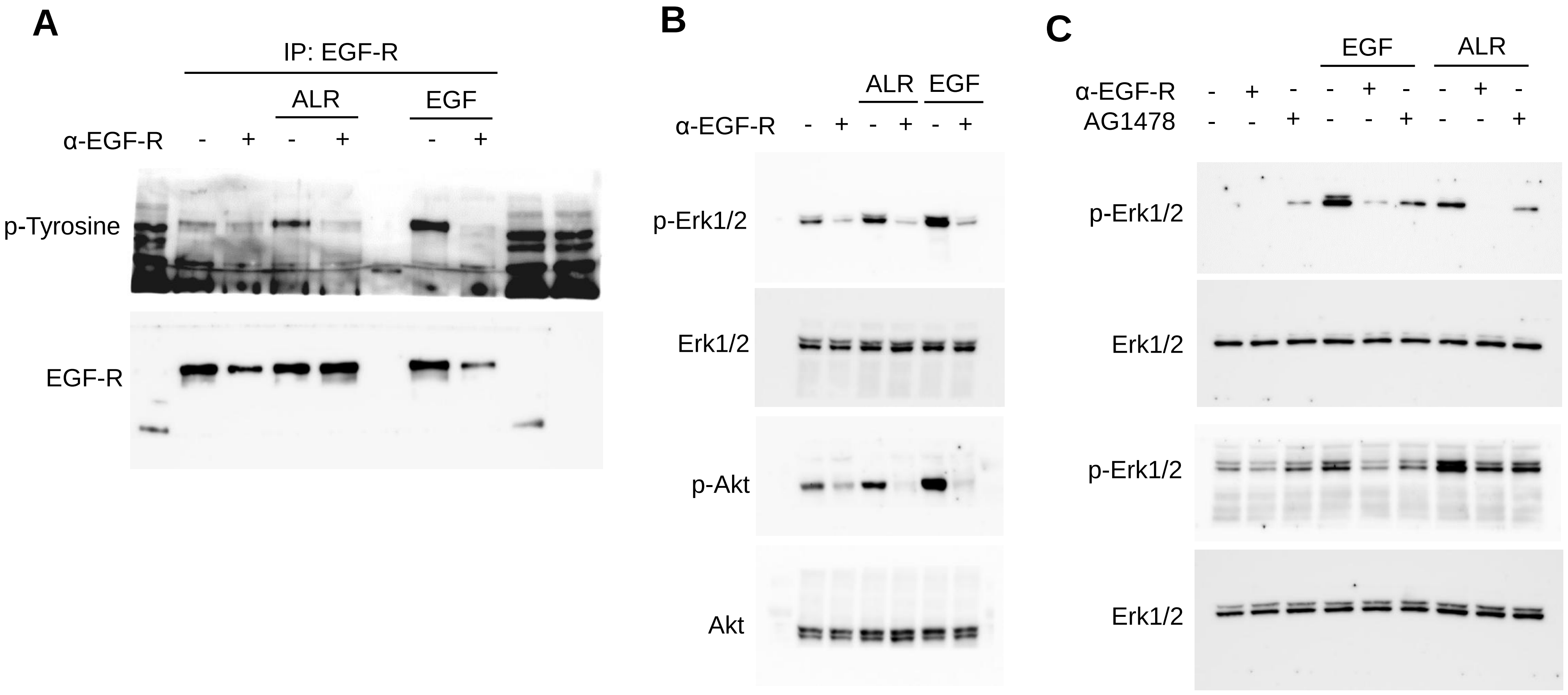

Fig. 2 uncropped blot images

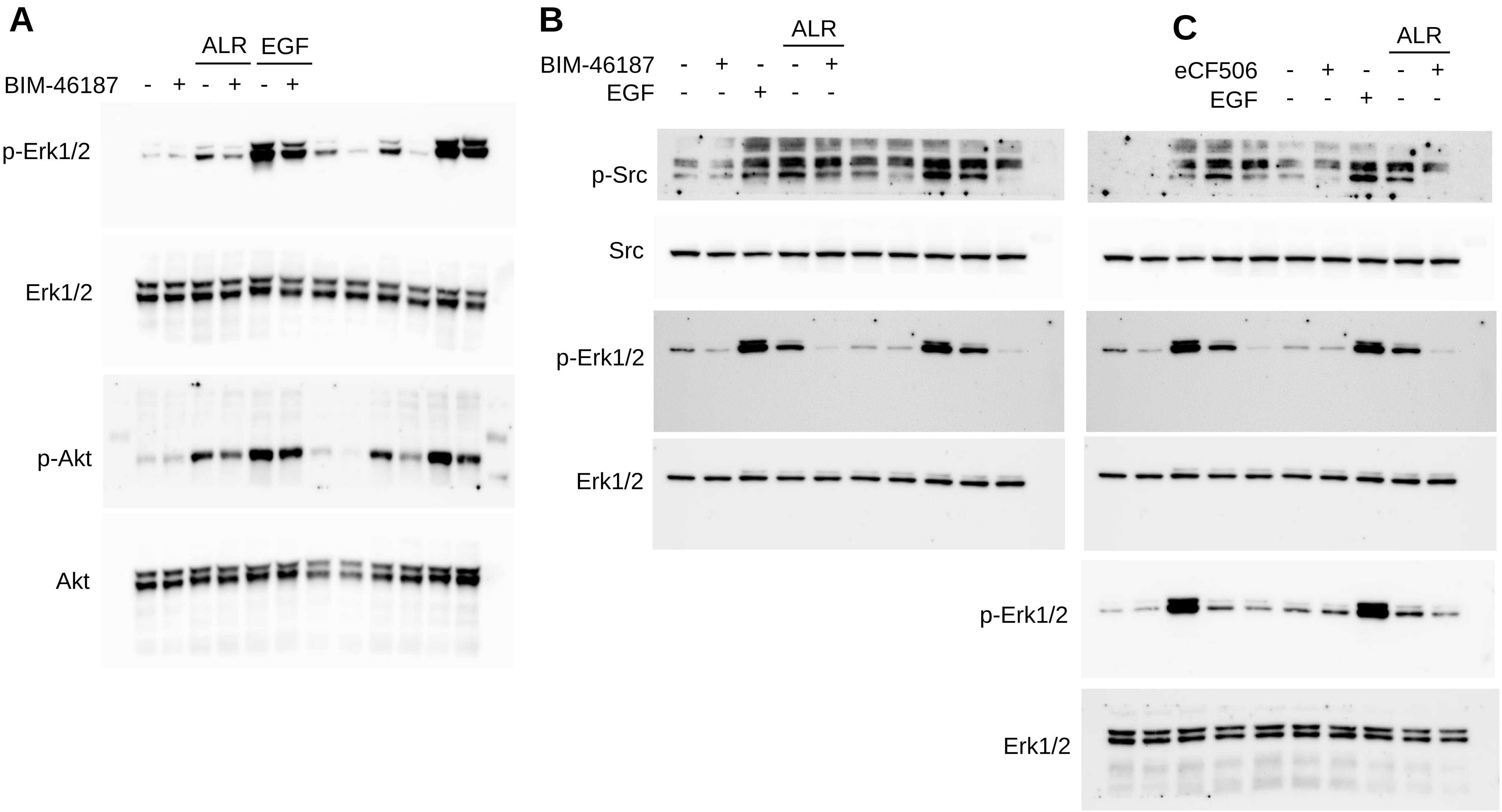

Fig. 3 uncropped blot images

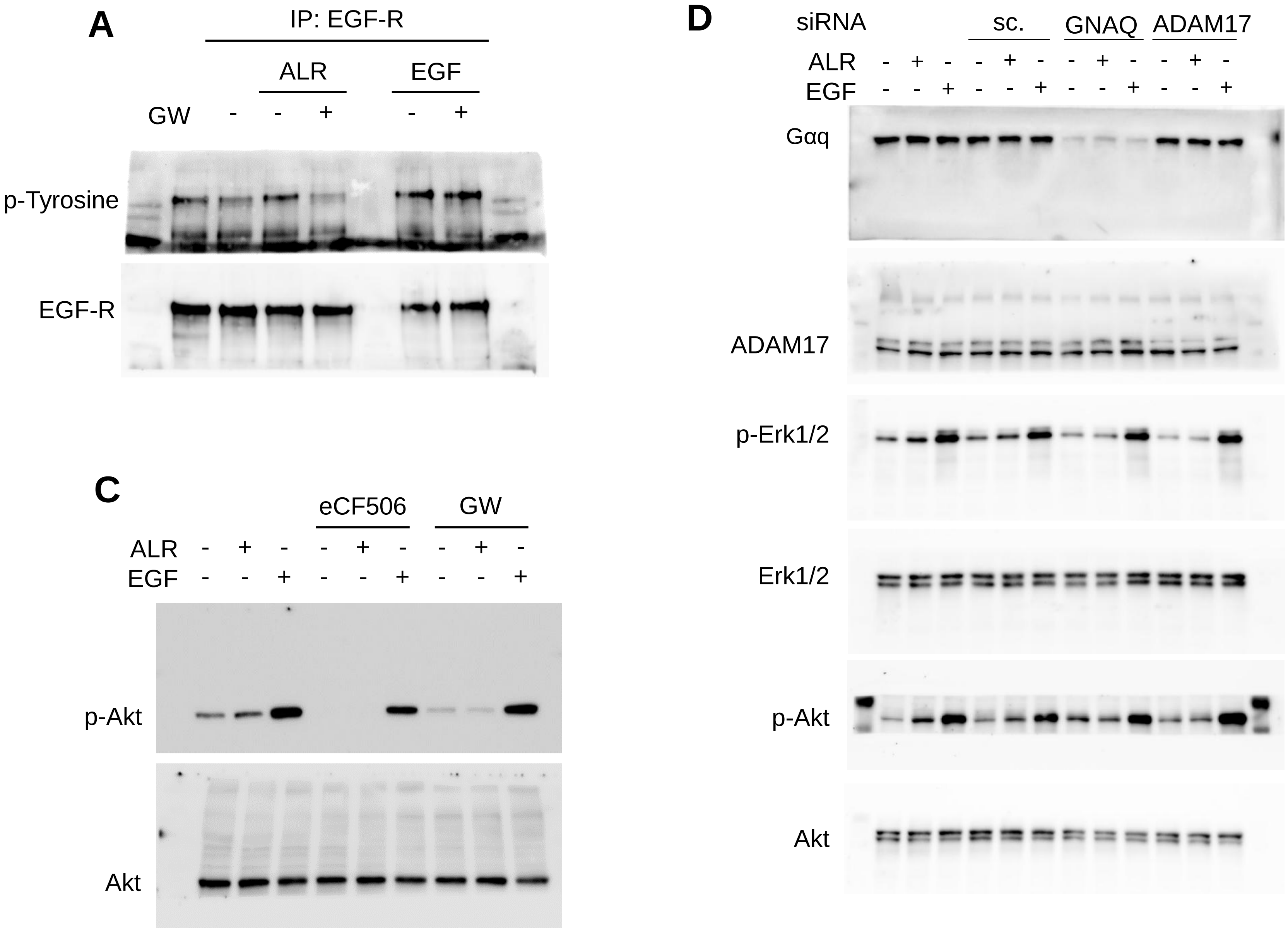

Fig. 3 uncropped blot images

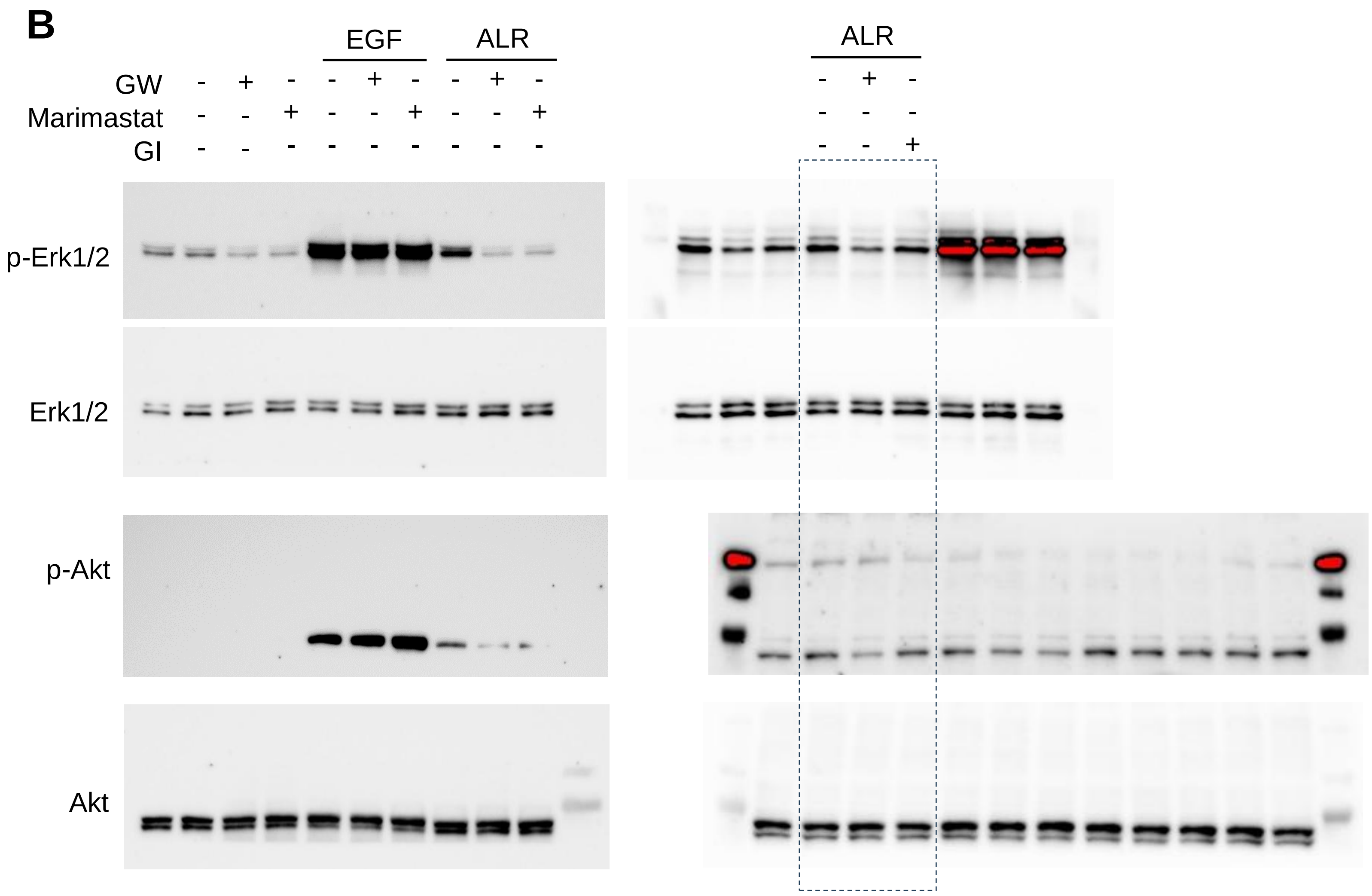

Fig. 3 uncropped blot images

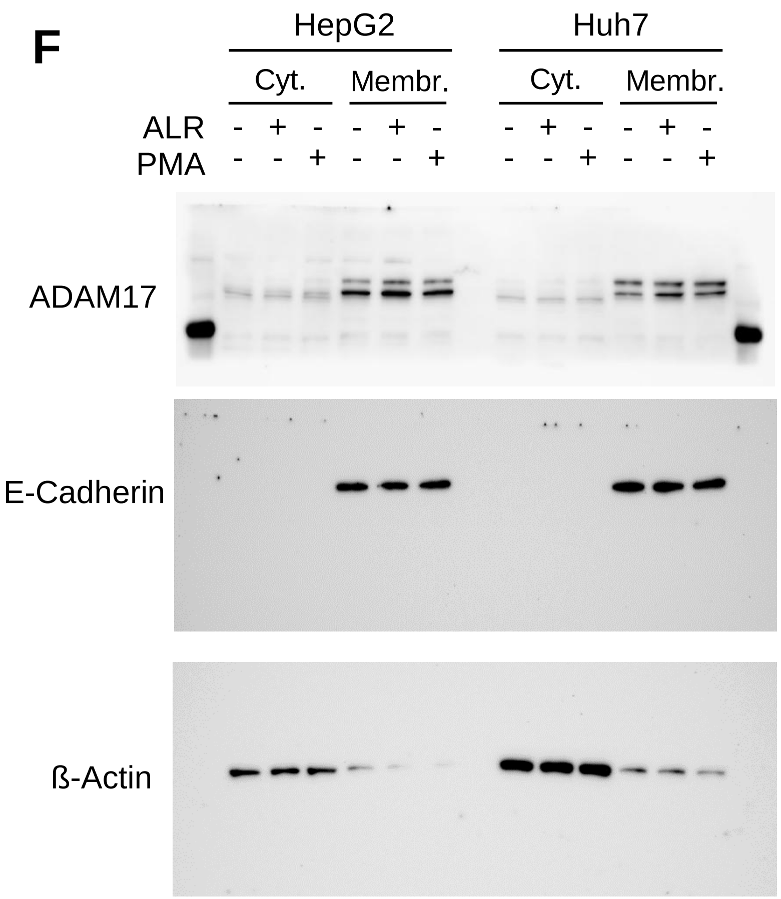

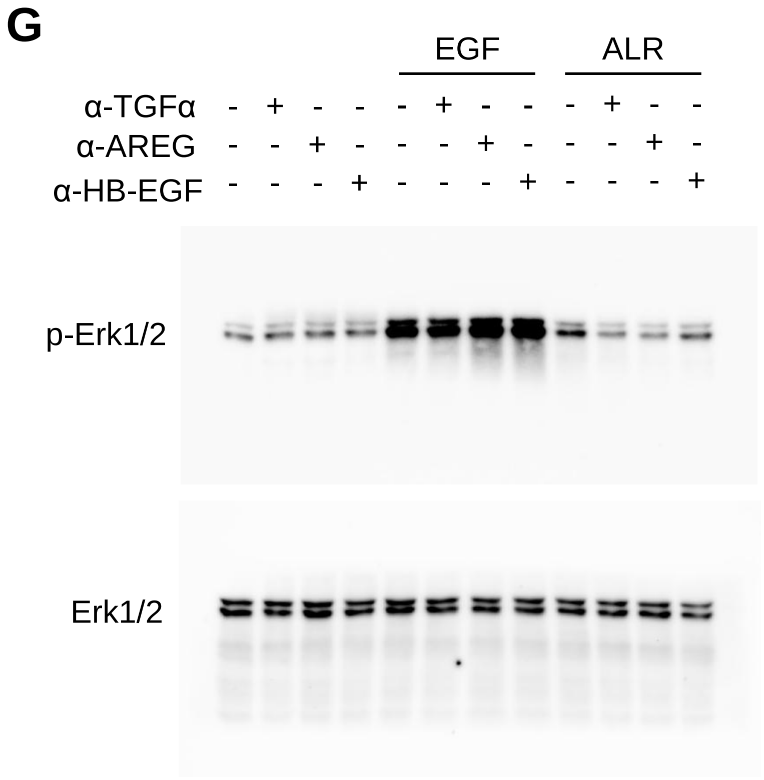

Fig. 4 uncropped blot images

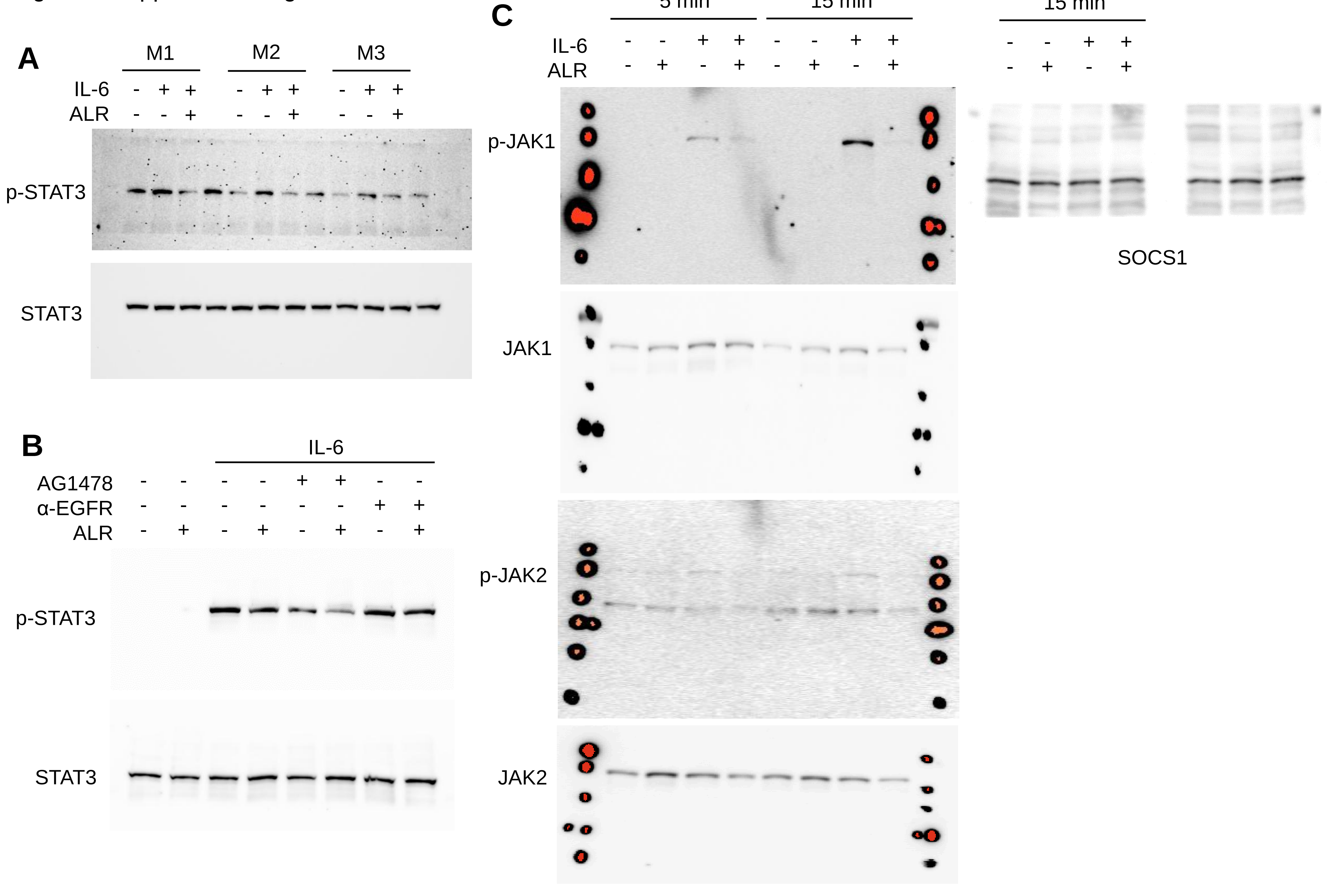

Fig. 4 uncropped blot images

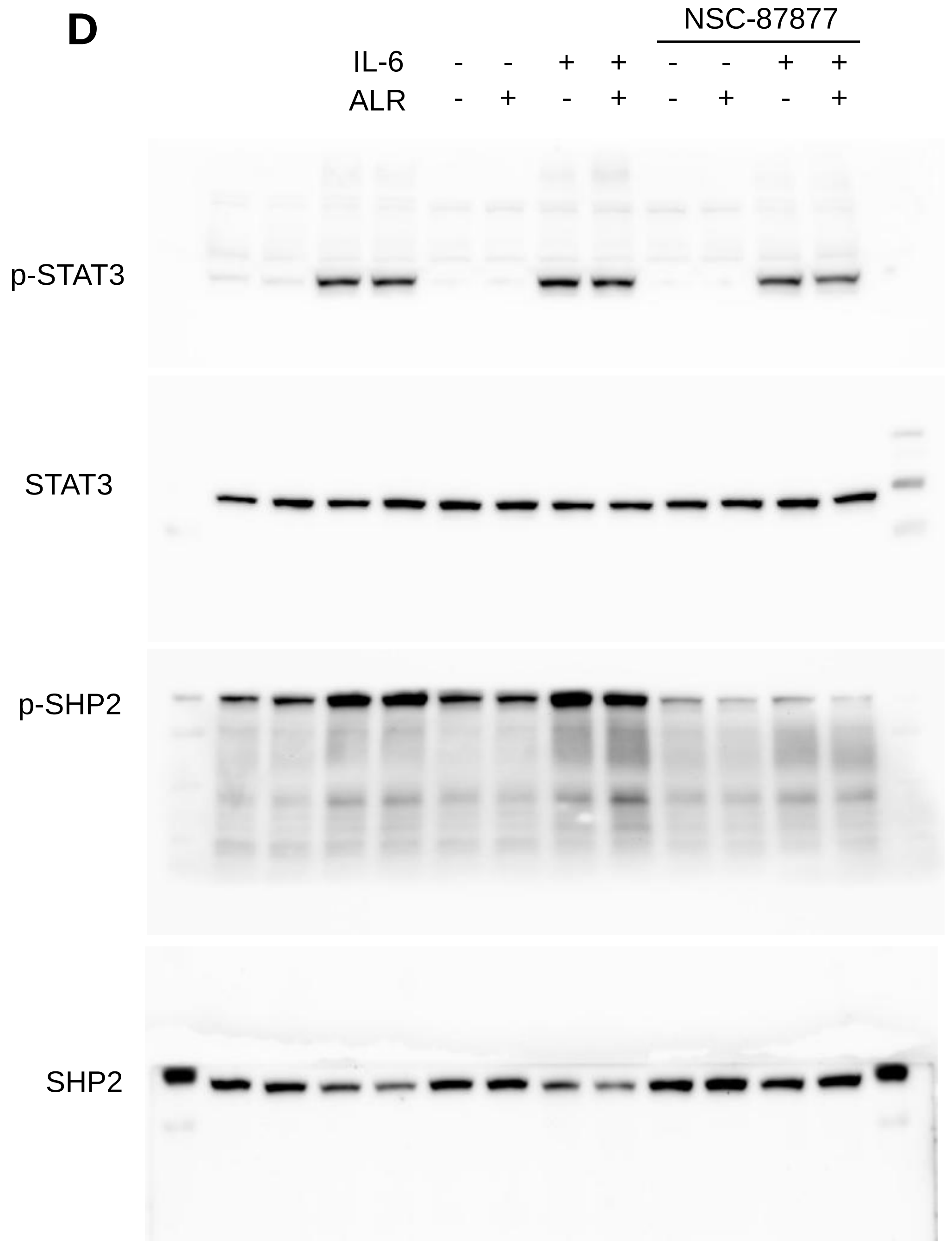

Fig. 4 uncropped blot images

D

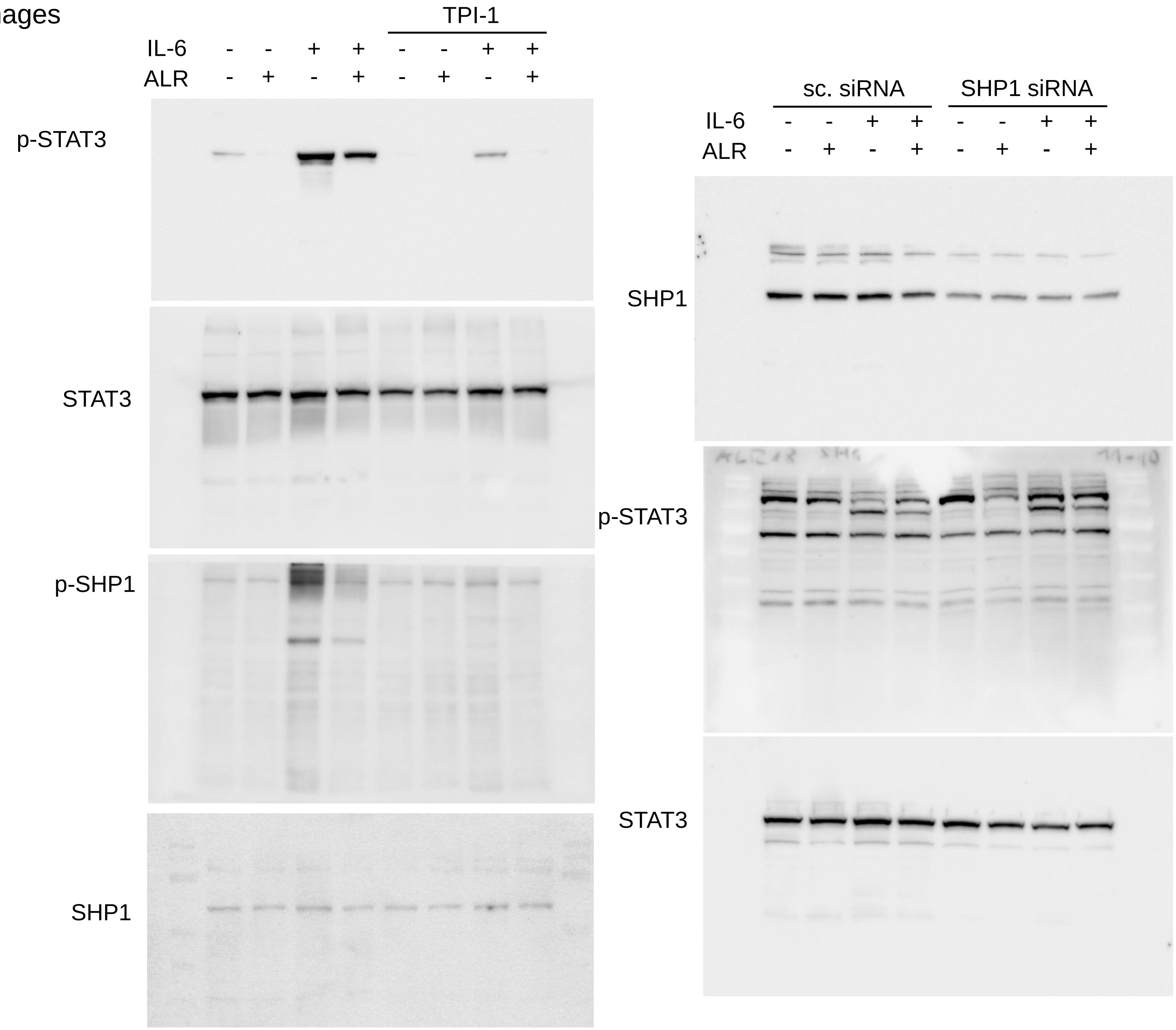

Fig. 5 uncropped blot images

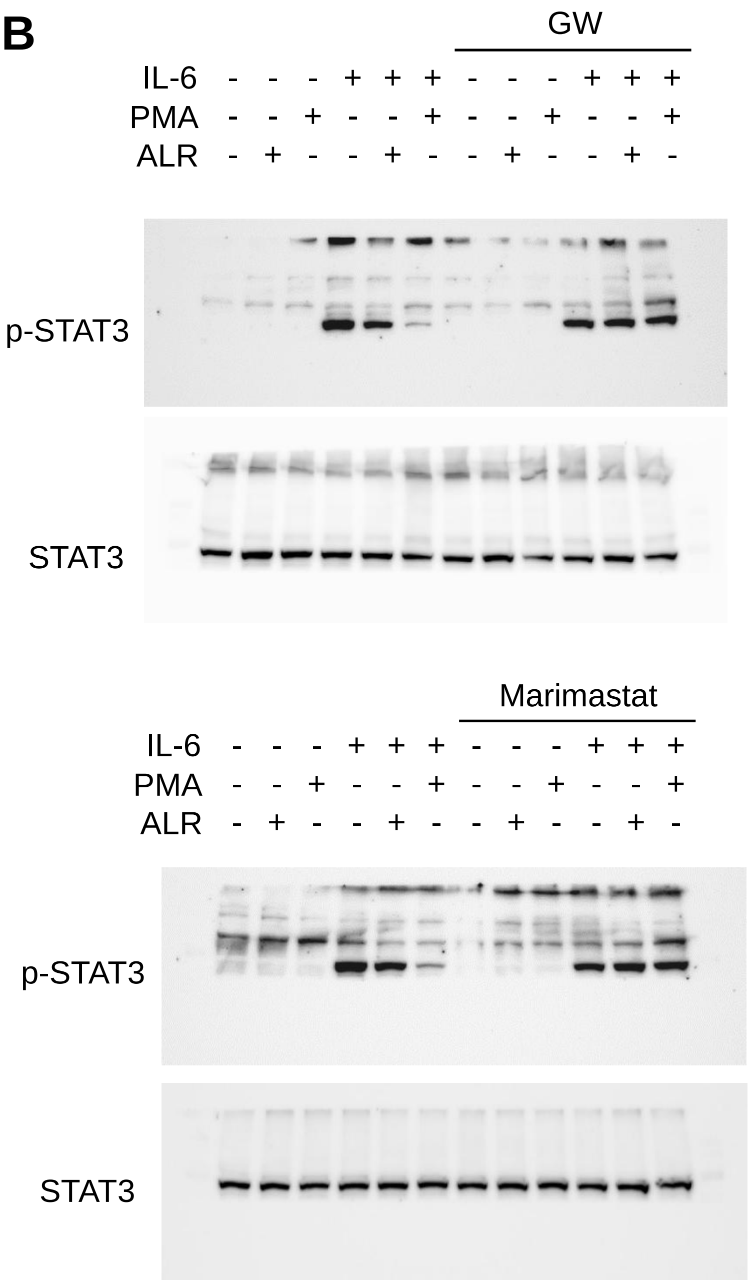

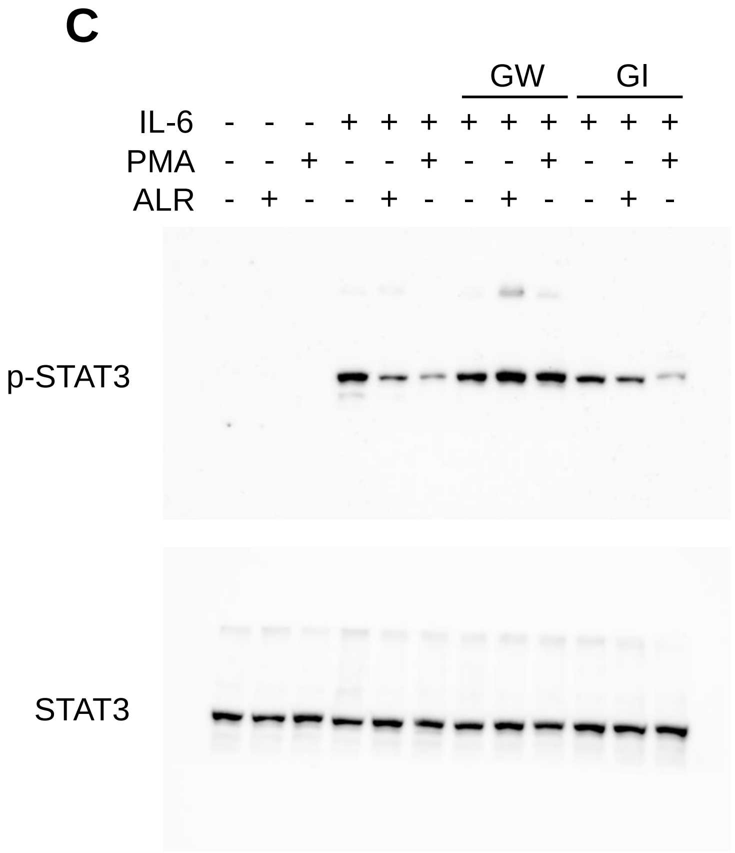

Fig. 5 uncropped blot images

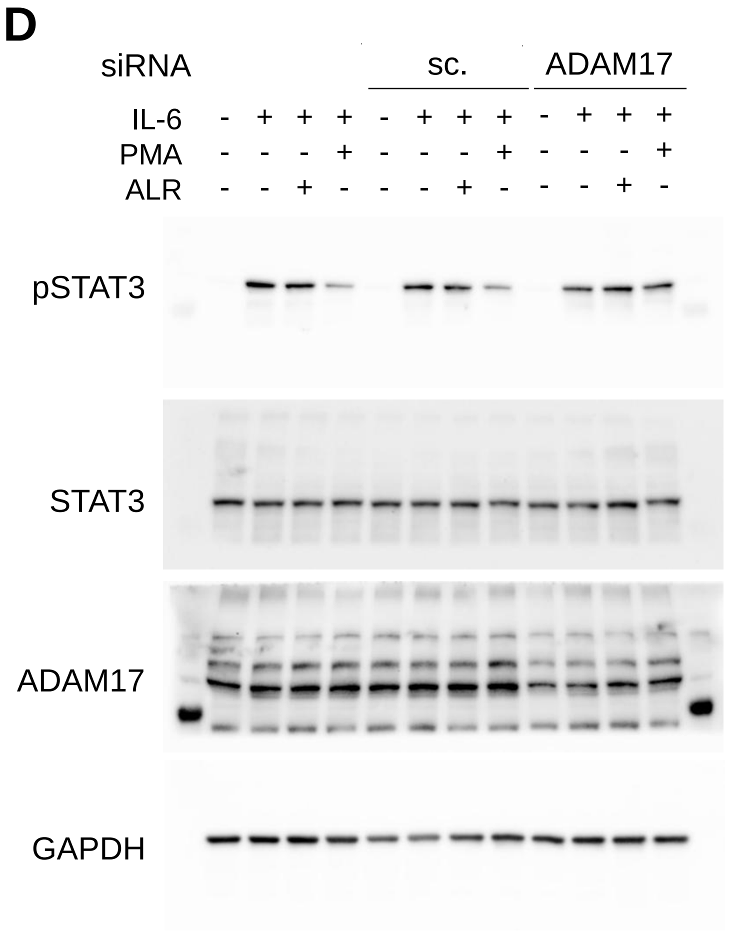

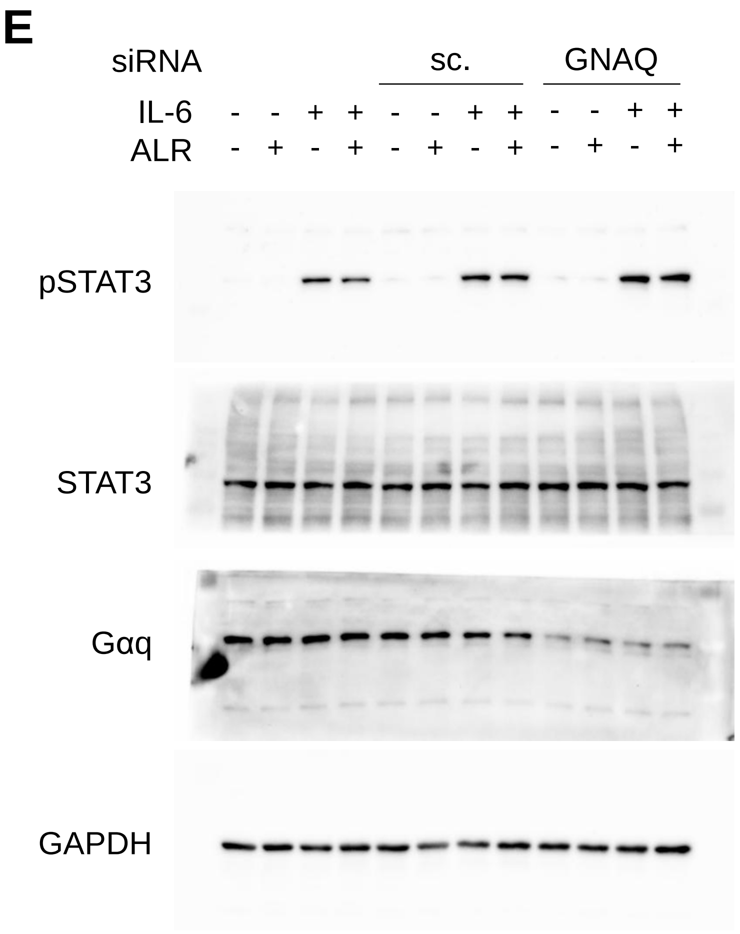

Fig. 5 uncropped blot images

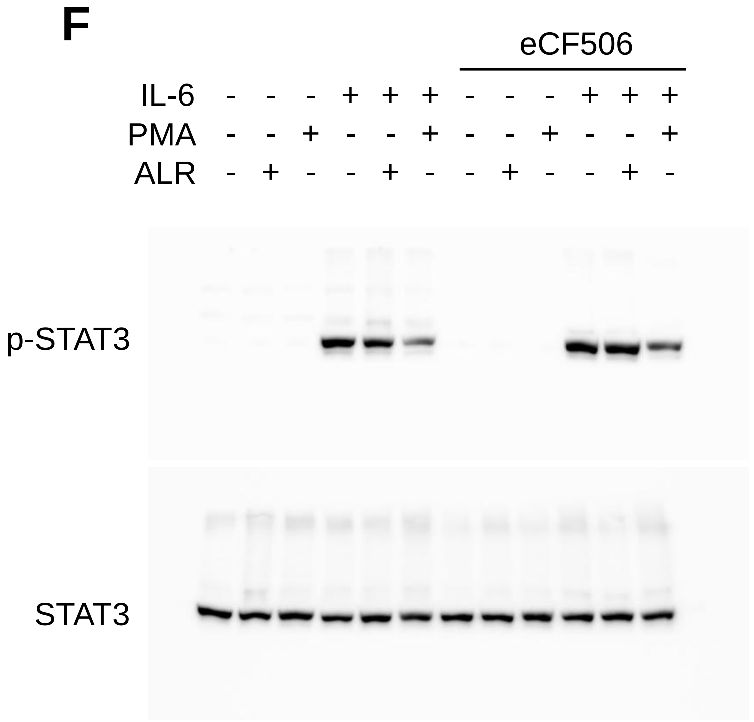

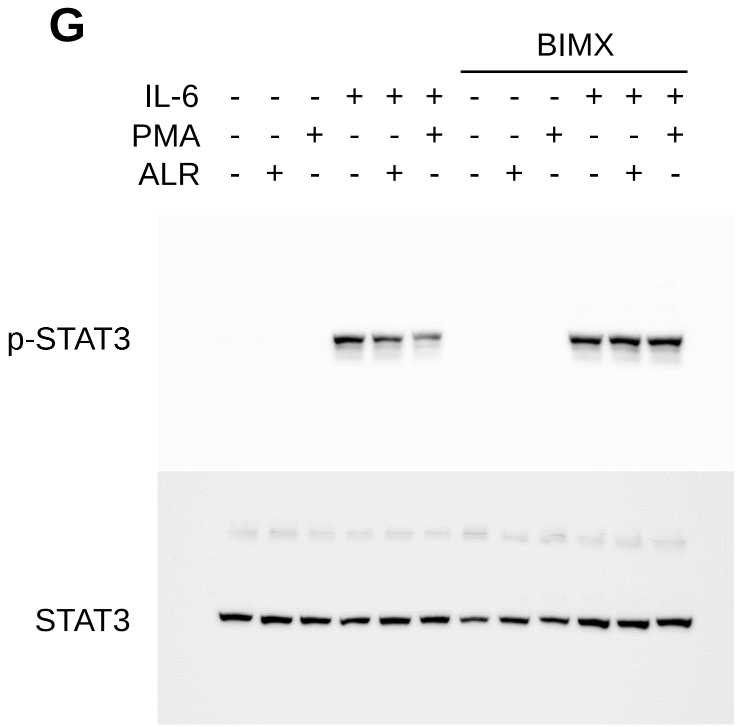

Fig. S1 uncropped blot images

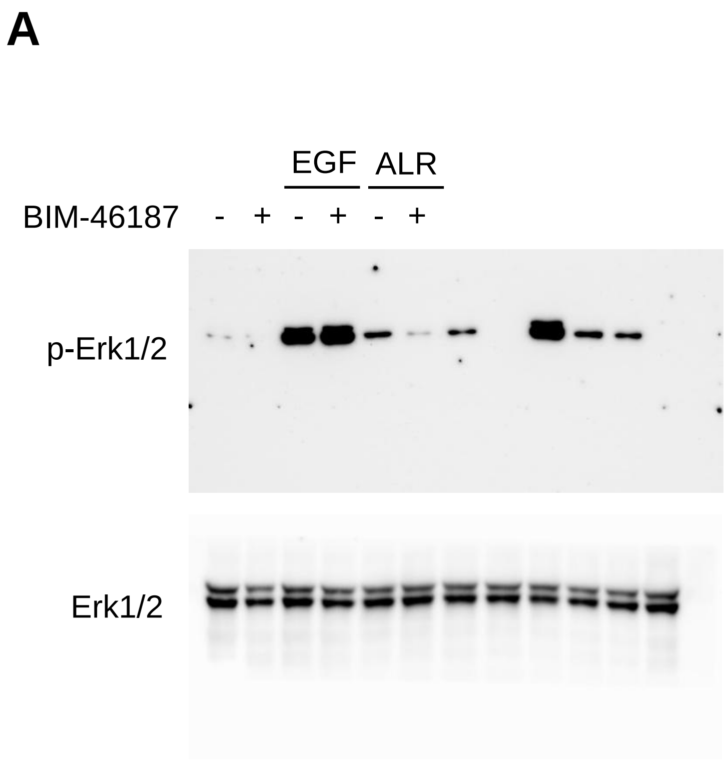

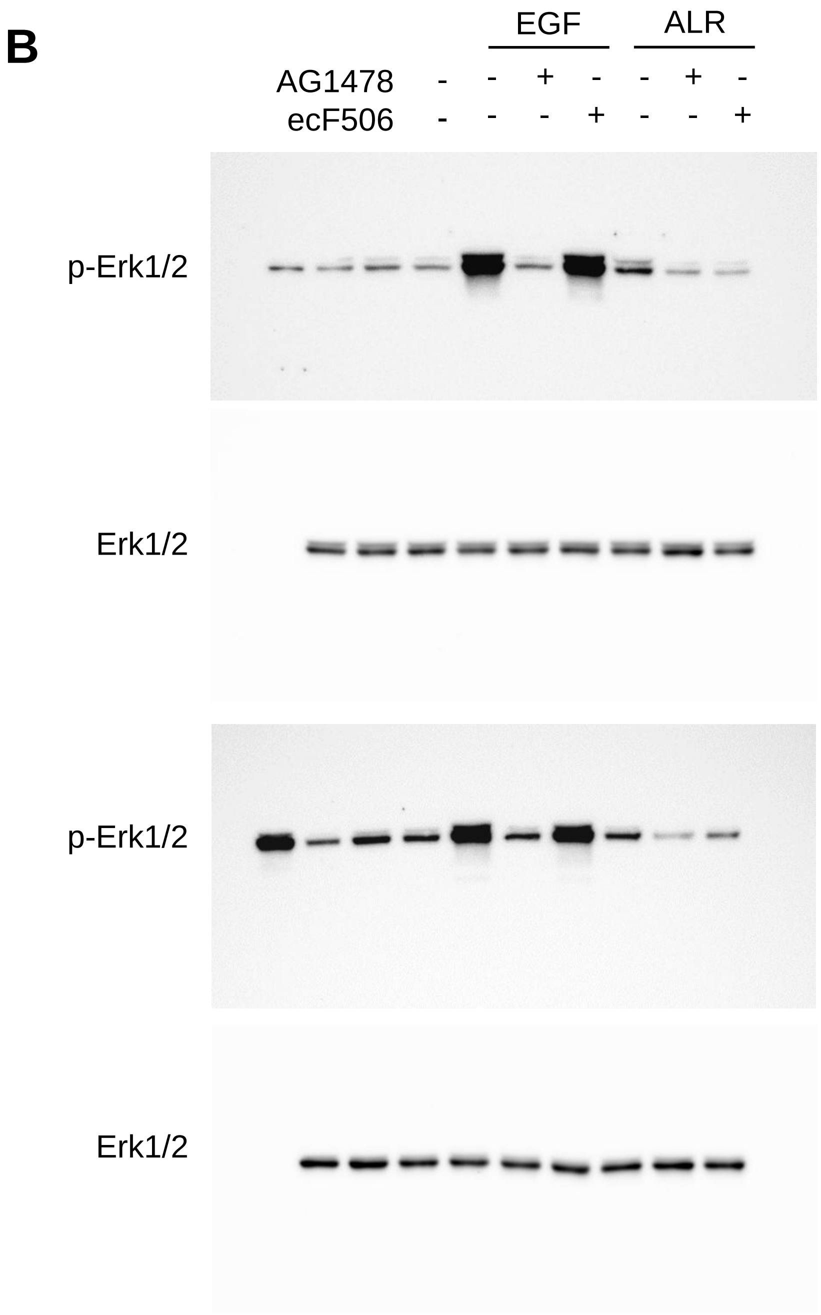

Fig. S3 uncropped blot images

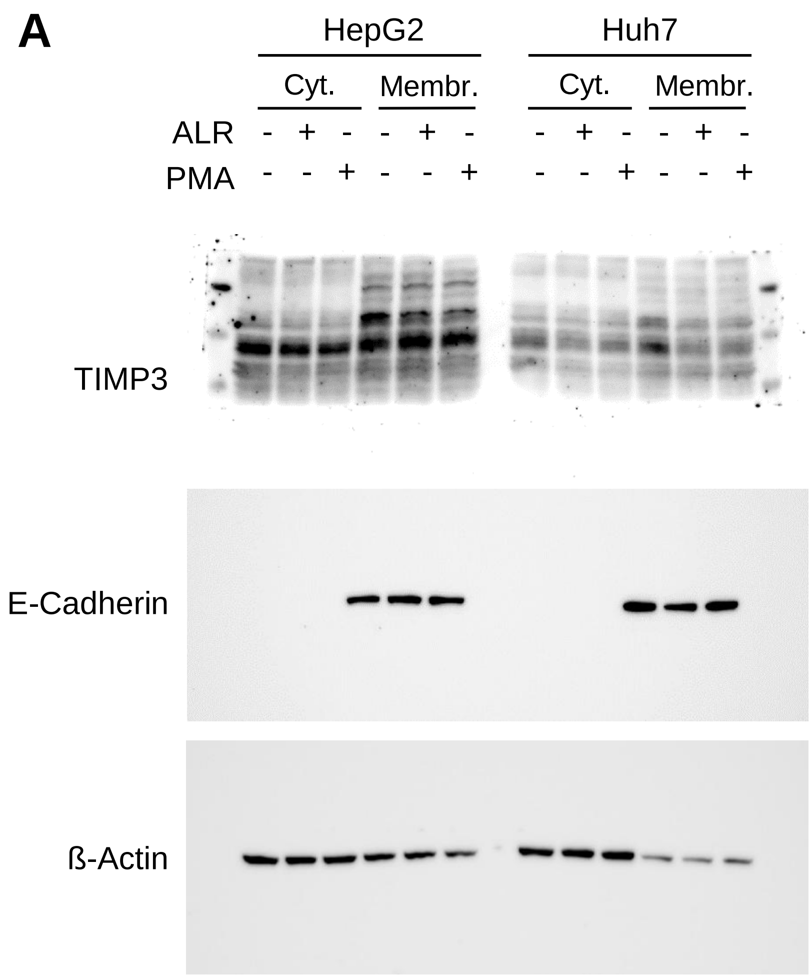

Fig. S3 uncropped blot images

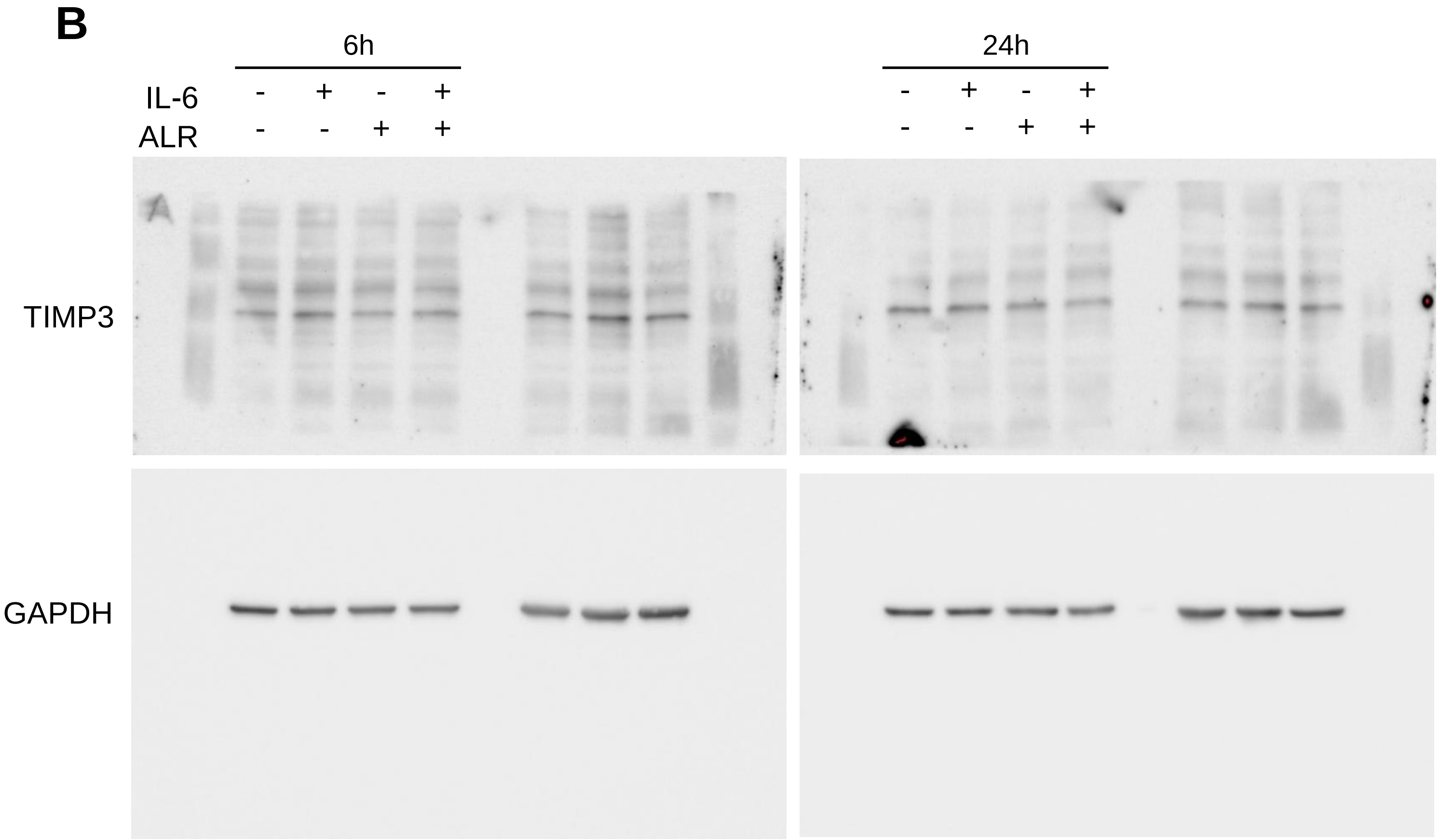

Fig. S4 uncropped blot images

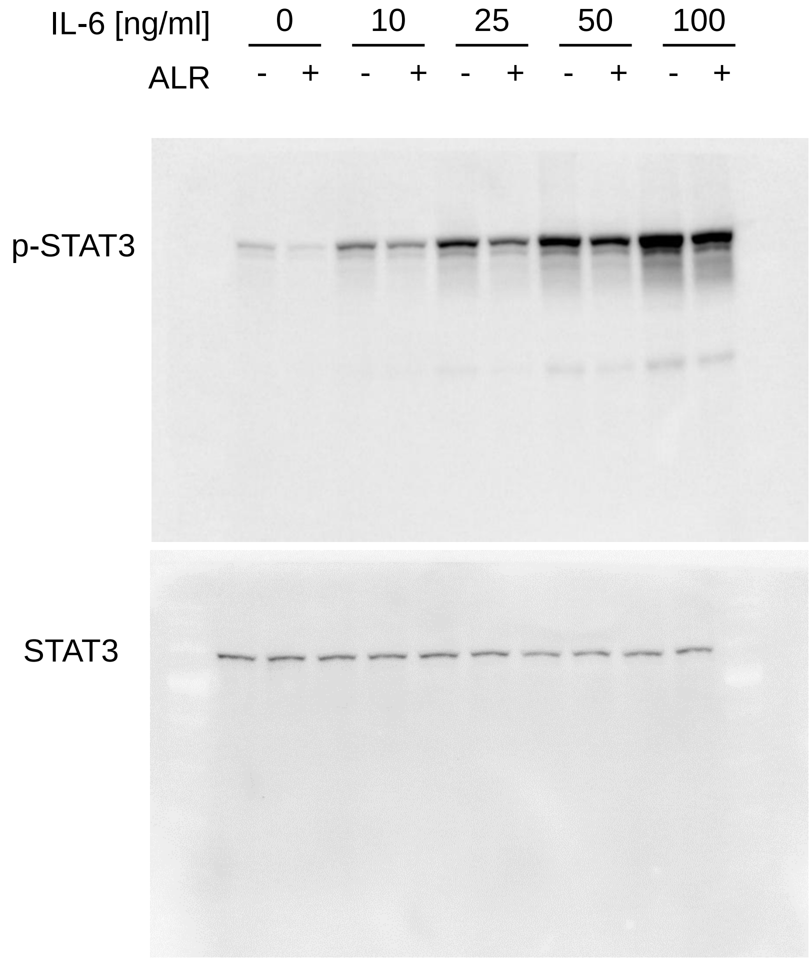

Fig. S5 uncropped blot images

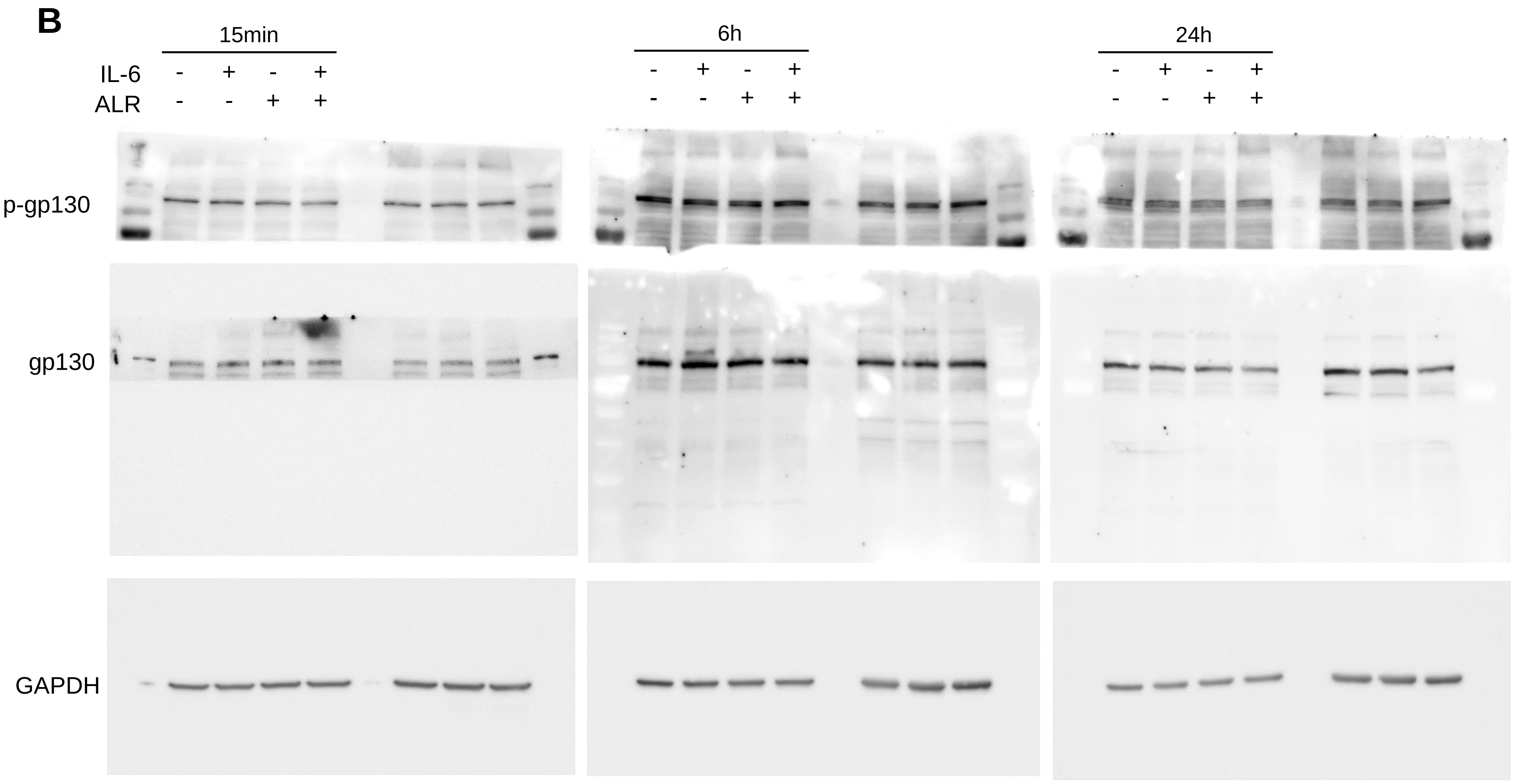

Fig. S6 uncropped blot images

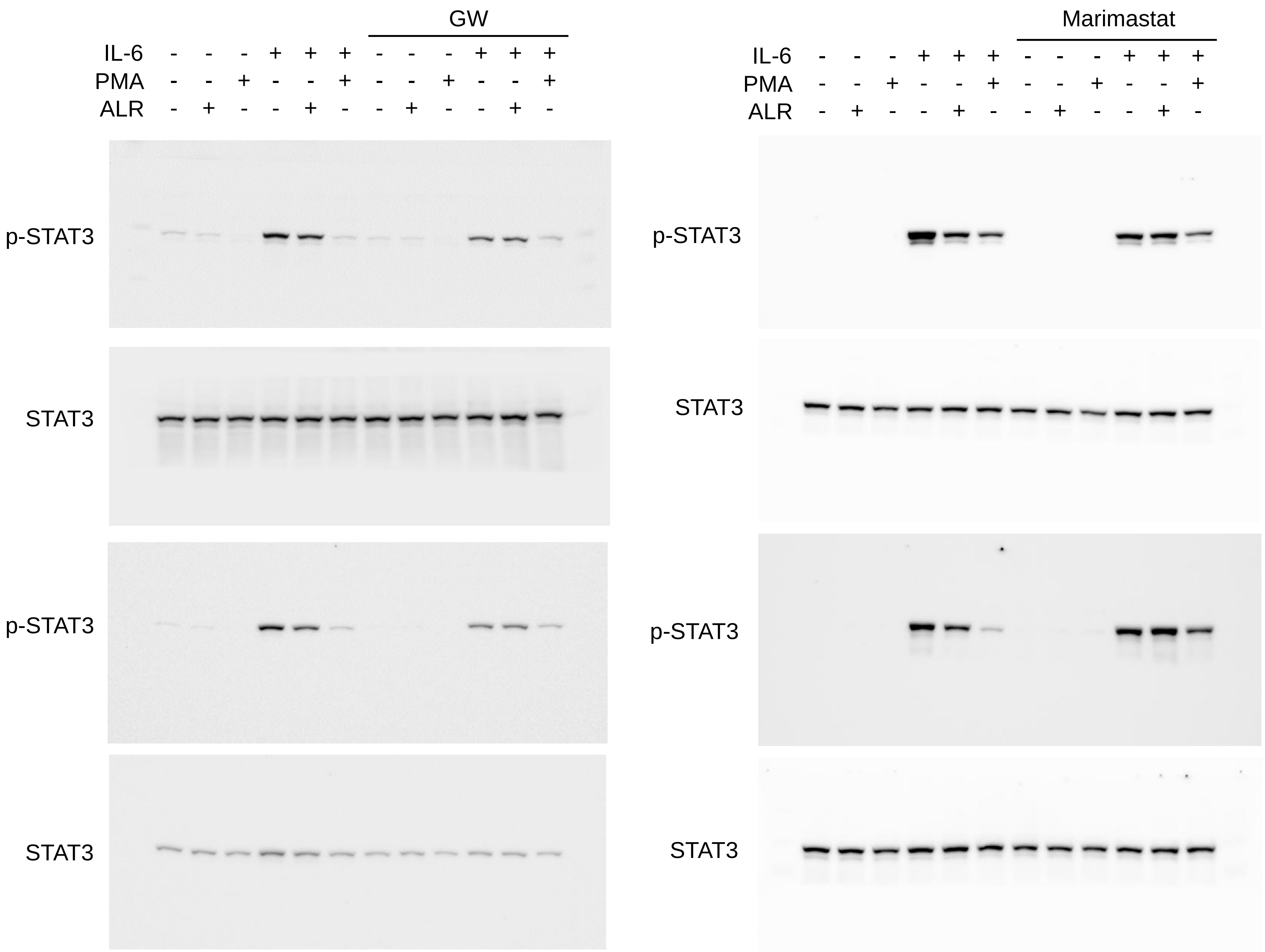

Supplement: Supplementary file 5 — Supplementary Material 5. [file 12964_2026_2782_MOESM5_ESM.pdf]
